# A mechanism-material-matrix framework for electrochemical advanced oxidation of persistent organic pollutants in wastewater

**DOI:** 10.1039/d6ra05751a

**Published:** 2026-08-24

**Authors:** Zhao Zhang, Guanghua Jing, Qiangqiang Lu, Ning Zhao, Zhikun Chen

**Affiliations:** a Xi'an Botanical Garden of Shaanxi Province (Institute of Botany Shaanxi Province) Xi'an Shaanxi 710061 China; b Key Laboratory of Soil Resource and Biotech Application, Shannxi Academy of Science Xi'an Shaanxi 710061 China zkchen1988@163.com

## Abstract

Persistent organic pollutants remain difficult to treat because disappearance of the parent compound can conceal incomplete mineralization, toxic intermediates, or high energy demand. This review asks when electrochemical advanced oxidation processes (EAOPs) provide defensible treatment rather than rapid analytical removal. We advance and test a mechanism-material-matrix-implementation framework: electrode and catalyst properties plus reactor geometry define the reactive-species window, while the wastewater matrix redistributes oxidant flux and changes both benefit and risk. Primary studies of anodic oxidation, electro-Fenton chemistry, active chlorine, sulfate-derived oxidants, three-dimensional cells, falling-film reactors, and reactive membranes are compared using initial concentration, removal kinetics, TOC or COD conversion, specific energy, by-product or toxicity evidence, and matrix transferability. The synthesis shows that flow-through and paired systems can combine high removal with substantial mineralization under defined conditions, whereas chloride-rich or adsorption-assisted systems can overstate performance when carbon balance and toxicity are omitted. Real-wastewater evidence further reveals trade-offs among matrix scavenging, oxyhalide formation, auxiliary energy, fouling, and durability. Four tables and nine figures convert these findings into mechanism, design, risk, and deployment criteria. The central conclusion is that EAOP performance is a system property, not an electrode property. This review is important because the framework turns heterogeneous literature into decision-ready criteria for selecting EAOPs as concentrate-destruction, pretreatment, post-treatment, or polishing stages.

## Abstract 2

We assess defensible EAOP treatment, not rapid analytical removal. A mechanism-material-matrix-implementation lens links electrodes, catalysts and reactor geometry to reactive-species windows; matrices redistribute oxidant flux, benefits and risks.

## Graphical abstract

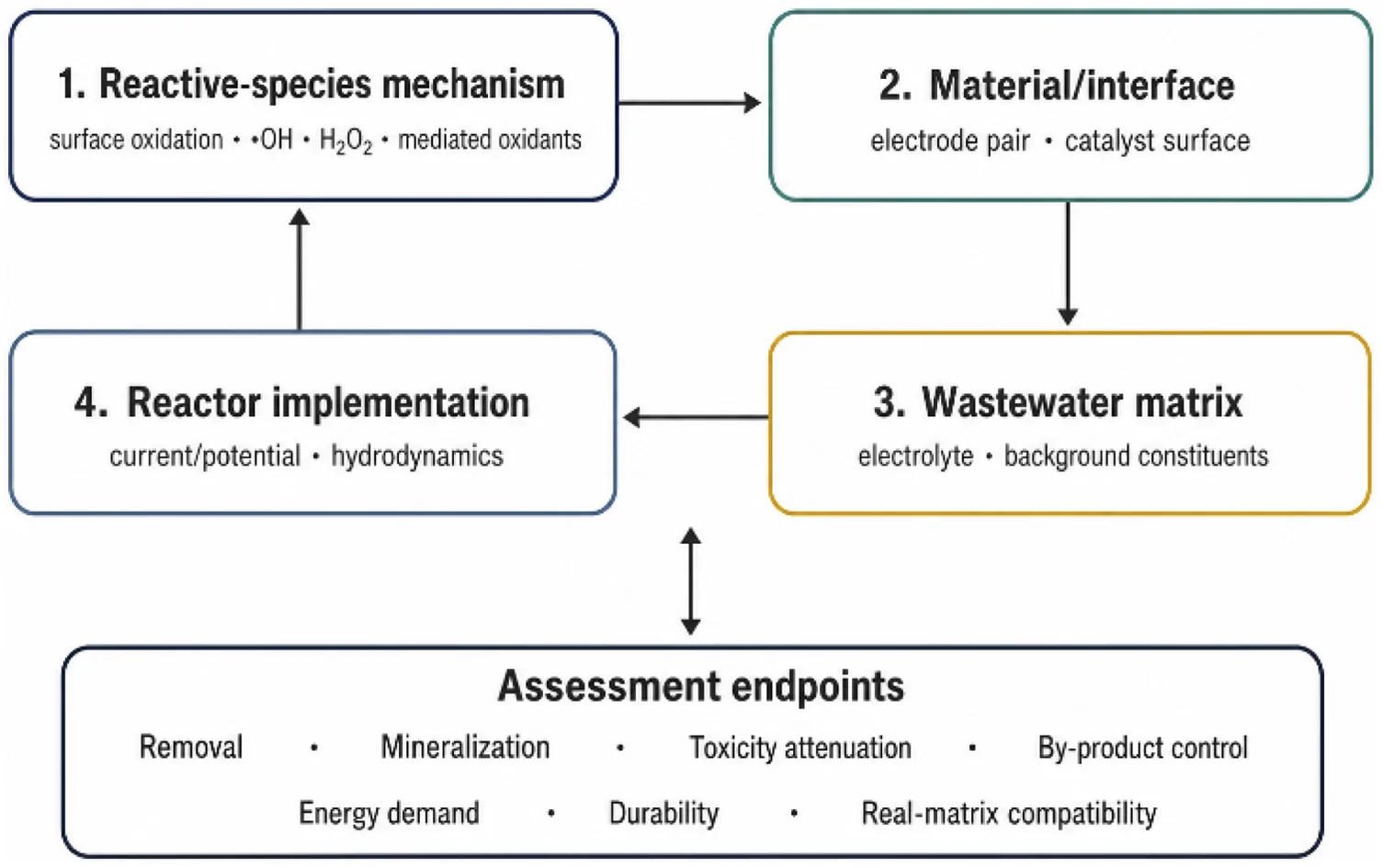

## 1. Introduction

Persistent organic pollutants and persistent emerging contaminants remain difficult targets for wastewater treatment because many of them resist biodegradation, pass through conventional treatment trains, or transform into products that retain biological activity. The practical problem is not limited to a single compound class. Pharmaceutical residues, dyes, phenols, pesticides, endocrine-disrupting compounds, halogenated industrial additives, and per- and polyfluoroalkyl substances (PFAS) differ in structure, water solubility, adsorption behaviour, and redox reactivity, but they share a common treatment difficulty: removal of the parent molecule does not necessarily prove mineralization, detoxification, or regulatory acceptability. Reviews of industrial effluent treatment have therefore shifted from simple disappearance metrics toward combined assessment of chemical oxygen demand, total organic carbon, toxicity, inorganic ion release, and operating cost.^1^ Recent work on by-product control in electrochemical oxidation also shows that a treatment process can improve one endpoint while creating another concern, especially in chloride-containing waters where chlorate, perchlorate, adsorbable organic halogens, or chlorinated intermediates may form.^2^

Electrochemical advanced oxidation processes (EAOPs) offer a compact route to generate oxidants *in situ* from water, dissolved oxygen, supporting electrolytes, or intentionally added precursors. In anodic oxidation, a high-overpotential anode such as boron-doped diamond (BDD), Magnéli-phase Ti_4_O_7_, PbO_2_, SnO_2_–Sb, mixed metal oxides, or dimensionally stable anodes can drive direct electron transfer and produce surface-bound hydroxyl radicals. In electro-Fenton systems, cathodic oxygen reduction generates H_2_O_2_, while Fe^2+^/Fe^3+^ cycling converts H_2_O_2_ into hydroxyl radicals; recent electro-Fenton reviews emphasize that cathode design, iron speciation, pH, and oxygen transfer control the apparent advantage of the process.^3^ Other EAOP variants add light, ultrasound, persulfate, peroxymonosulfate, membranes, adsorption, or biological pretreatment. These additions can expand the oxidant portfolio, but they also complicate attribution because parent-compound decay may arise from adsorption, coagulation, active chlorine, sulfate radicals, direct anodic oxidation, or homogeneous radical chemistry in the same reactor.

**Fig. 1:**
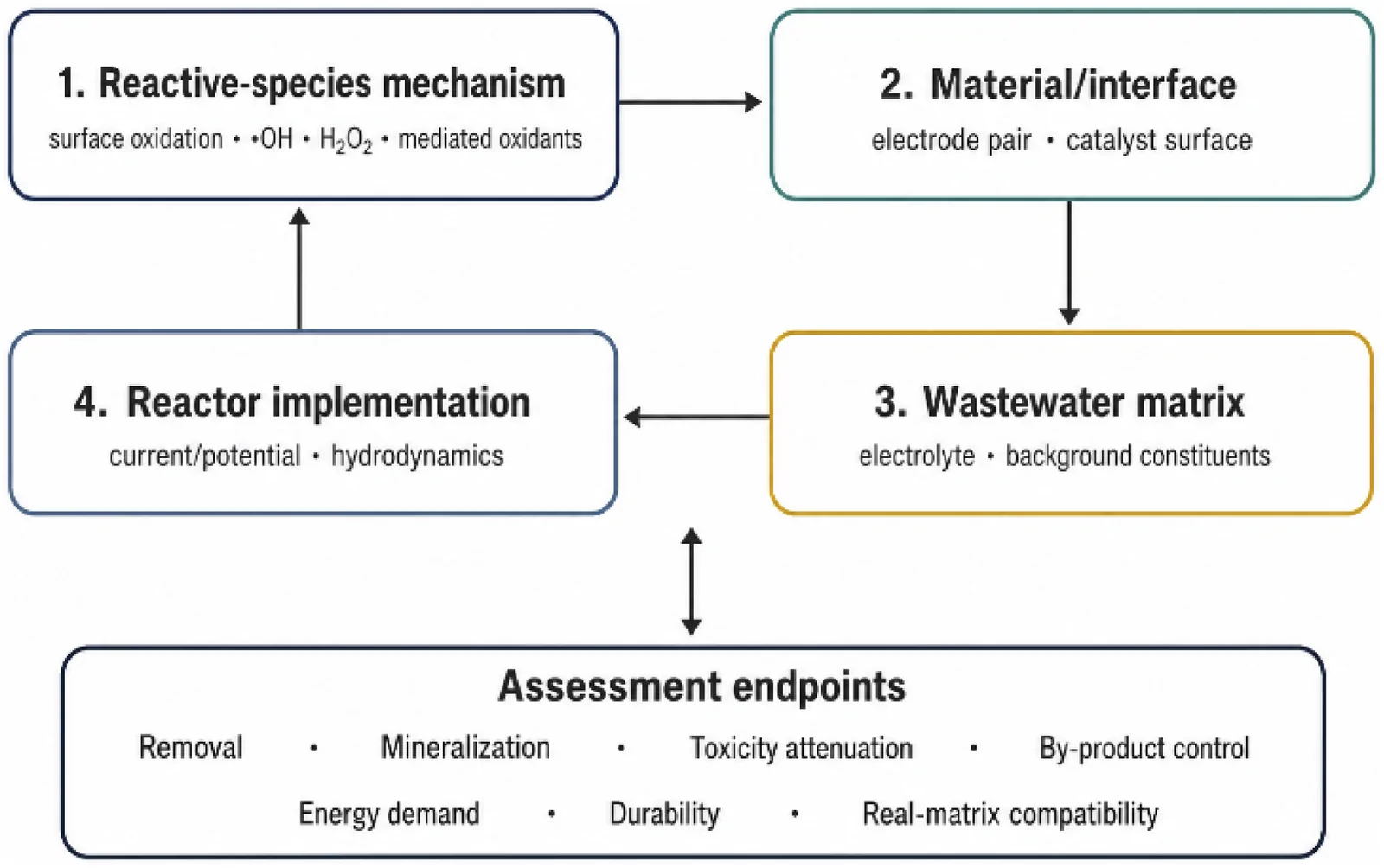
Coupled mechanism-material-matrix-implementation framework for EAOP treatment of persistent organic pollutants. The framework treats performance as a feedback-controlled system and evaluates every configuration against removal, mineralization, toxicity, by-product, energy, durability, and real-matrix endpoints.

The central argument tested throughout this review is that EAOP performance is not an intrinsic property of an electrode. It emerges from alignment among four coupled levels: (i) the reactive-species mechanism, (ii) the electrode or catalyst interface, (iii) the water matrix, and (iv) reactor implementation. Fig. 1 makes this proposition explicit. Applied current or potential, electrode pairing, hydrodynamics, electrolyte composition, and wastewater background set the balance among surface oxidation, hydroxyl-radical chemistry, H_2_O_2_-mediated pathways, sulfate- or chlorine-derived oxidants, and non-oxidative removal. That balance is then evaluated against a common endpoint set: parent-compound removal, mineralization, toxicity attenuation, transformation-product control, energy demand, durability, and real-matrix compatibility. The feedback arrows are essential: a higher current can improve conversion while increasing energy and oxychlorine formation, whereas a lower current can reduce those risks but leave persistent intermediates.

The evidence base is sufficiently broad for quantitative comparison but remains uneven. Some studies report kinetics, TOC removal, energy, toxicity, and transformation products together, whereas others report only parent decay in a synthetic matrix. In a direct comparison of ozone, anodic oxidation, electro-Fenton, and coupled electro-Fenton/anodic oxidation, complete parent removal did not translate into comparable mineralization in municipal effluent.^4^ Likewise, rapid 4-ethylphenol loss in saline water coincided with residual organic carbon and chlorinated intermediates.^5^ These contrasts establish the review's evaluative premise: apparent removal must be interpreted with treatment depth, energy, and risk.

The scope spans general electrochemical and industrial-effluent treatment,^1,6–8^ BDD and anode-centred oxidation,^9–16^ electro-Fenton and cathodic oxidant generation,^17–25^ reactor, membrane, and hybrid process intensification,^26–37^ sector-specific wastewater and treatment-train evidence,^38–51^ and adjacent pollutant-class studies used only to interpret transferability rather than as direct EAOP benchmarks.^52–60^ Recent literature requested during peer review reinforces the need to separate destructive oxidation from adjacent removal routes. Waste-derived peroxydisulfate activators and regenerable biochar catalysts extend reactive-species control beyond conventional electrodes,^61,62^ while current reviews of pharmaceutical AOPs and MOF adsorption show how catalyst-assisted degradation, separation, and polishing can be combined without treating adsorption as mineralization.^63,64^

Unlike reviews organized primarily by electrode class or removal percentage, this review follows a causal sequence from reactive species to interface, matrix, reactor, pollutant class, and deployment role. Its objectives are to (i) test the four-level coupling proposition against quantitative primary evidence, (ii) compare studies through a standardized endpoint scorecard, and (iii) define where EAOPs fit within wastewater treatment trains. The analysis targets four recurrent research gaps: incompatible normalization, insufficient real-matrix validation, incomplete by-product and toxicity accounting, and weak reporting of auxiliary energy and long-term durability.

## 2. Chemical and interfacial mechanisms in EAOPs

Section 2 establishes the first part of the central argument by moving from interfacial charge transfer to bulk oxidant generation, then to matrix competition and by-product formation. This sequence shows how material choice acquires meaning only after reactive species and the chemical environment are connected to measurable treatment endpoints.

### 2.1 Direct anodic oxidation and surface-bound oxidants

The mechanistic identity of an EAOP is determined first by the interface at which charge transfer, radical formation, and oxygen evolution compete. Direct anodic oxidation can occur when an adsorbed pollutant exchanges electrons with the anode surface, but this route is usually limited by mass transfer and surface coverage for trace organic pollutants. The broader value of anodic oxidation comes from surface-bound oxidants, especially physisorbed hydroxyl radicals on non-active anodes such as boron-doped diamond (BDD) and Magnéli-phase titanium oxides. These materials bind hydroxyl radicals weakly enough for the oxidant to behave as a high-potential, poorly selective species near the interface, whereas active anodes such as RuO_2_- or IrO_2_-based dimensionally stable anodes (DSA) tend to form chemisorbed oxygenated states that favor partial conversion and oxygen evolution. This distinction is not absolute, because crystal defects, oxygen vacancies, hydrodynamic residence time, and solution composition all shift the local availability of reactive oxygen species.

Quantitative comparisons illustrate why the non-active/active-anode classification is useful but insufficient as a design rule. In the degradation of norfloxacin, da Silva *et al.*^65^ reported apparent rate constants of 0.75 h^−1^ in synthetic sulfate medium, 0.82 h^−1^ in sulfate-amended real wastewater, and 0.45 h^−1^ in unamended real wastewater, showing that the same BDD/DSA comparison is strongly conditioned by conductivity and matrix composition. The study also found that Nb/BDD achieved similar current efficiency with about 60% lower energy demand than Si/BDD, which separates anode chemistry from the electrical penalty imposed by substrate resistance.^65^ For methyl orange, Isarain-Chavez *et al.*^66^ found that mixed-oxide anodes reached 96–98% color removal over 240 min, while Ti/RuO_2_ reached only 76–85% across 7.1–70 mA cm^−2^; the best Ti/Ir–Pb configuration gave 76.0% TOC removal, but the increase in current density also raised cell voltage from 4.5 to 8.1 V. Such results support a practical conclusion: electrode screening based on parent-compound disappearance alone can overstate process value when mineralization, voltage, and long-lived intermediates are not considered.

The most compelling recent evidence comes from flow-through reactive electrochemical membranes, where the transport field is deliberately coupled to the reactive interface. Kang *et al.*^67^ showed that a 7 µm porous Ti reactive electrochemical membrane coated with defective TiO_2_ nanosheets removed 94.0% of 4-chlorophenol in 13.6 s, whereas a 105 µm membrane removed 67.4% under the same residence time; TOC removal after 54.5 s was 96% and 64%, respectively. The reported apparent rate constant increased from 4.6 to 18.9 min^−1^ as the pore scale decreased, while the energy consumption for 90% removal fell from 2.95 to 0.86 kWh m^−3^.^67^ These values show that mechanism is not only a property of the anode material. It is also a property of how the reactor confines diffusion layers and prevents oxidants from being lost to oxygen evolution or bulk scavenging.

### 2.2 Reactive-species generation, interconversion, and radical pathways

Beyond the anode surface, EAOPs generate a family of oxidants whose selectivity and lifetime differ substantially. Electro-Fenton systems reduce dissolved oxygen to H_2_O_2_ at carbonaceous cathodes and then activate H_2_O_2_ with Fe^2+^ or heterogeneous iron sites to produce hydroxyl radicals in the bulk or at catalyst surfaces. This pathway often improves mineralization because oxidants can attack intermediates away from the anode, but it commonly requires acidic pH, oxygen transfer, iron control, and management of sludge or catalyst stability. For malachite green, Teymori *et al.*^68^ reported complete color removal in 15 min and 95.3% TOC removal within 30 min at pH 3 with iron electrodes and H_2_O_2_; the electro-Fenton apparent rate constant was 0.092 min^−1^, compared with 0.020 min^−1^ for electrocoagulation. The same work showed that performance declined from complete removal at up to 1000 mg L^−1^ dye to 92.3%, 80.1%, and 65.0% at 1500, 2000, and 3000 mg L^−1^ within 30 min, which is a useful reminder that radical demand increases faster than design intuition at high organic load.^68^

Representative elementary steps clarify where these pathways originate. Anodic water discharge forms surface-bound hydroxyl radicals, the two-electron oxygen-reduction reaction produces H_2_O_2_ at the cathode, Fe^2+^ activates H_2_O_2_ in electro-Fenton systems, persulfate activation yields sulfate radicals, and chloride oxidation generates active chlorine. The dominant route depends on electrode potential, catalytic sites, oxygen transfer, pH, and electrolyte composition.^8,9^M + H_2_O → M(˙OH) + H^+^ + e^−^O_2_ + 2H^+^ + 2e^−^ → H_2_O_2_Fe^2+^ + H_2_O_2_ → Fe^3+^ + ˙OH + OH^−^S_2_O_8_^2−^ + e^−^ → SO_4_^2−^ + SO_4_˙^−^

For chloride-containing waters, 2Cl^−^ → Cl_2_ + 2e^−^ followed by Cl_2_ + H_2_O ⇌ HOCl + H^+^ + Cl^−^. HOCl/OCl^−^ can accelerate parent loss, but further anodic oxidation can produce chlorate and perchlorate. These reactions explain why reactive-species identification must be coupled to speciation, mass transfer, and by-product monitoring rather than inferred from scavenger tests alone.

Heterogeneous electro-Fenton and paired-electrode systems attempt to retain the oxidative strength of Fenton chemistry while reducing the burden of soluble iron. Xia *et al.*^69^ used Fe single atoms coordinated on N-doped carbon in a gas-diffusion electrode system and achieved complete degradation of 0.14 mM 2,4-dichlorophenol within 90 min, whereas electro-generated H_2_O_2_ alone removed 17.3% and adsorption removed 15.6%. The same study reported 67.6% mineralization after 300 min with BDD/air-diffusion heterogeneous electro-Fenton, demonstrating that coupling cathodic H_2_O_2_ formation with anodic oxidation can convert parent removal into deeper oxidation.^69^ In a related but chemically distinct strategy, Li *et al.*^70^ reported an electro-enhanced peroxymonosulfate process in which a Fe single-atom cathode gave a bisphenol A rate constant of 0.073 min^−1^, compared with 0.015 min^−1^ at the anode, and maintained 99.5–97.5% removal over 17 h of continuous operation. The energy efficiency was favorable at 0.15 kWh log^−1^ m^−3^, but the interpretation depends on the cost and downstream fate of peroxymonosulfate as well as catalyst durability.^70^

Halogen-mediated oxidation is mechanistically different because chloride or bromide can convert an electrochemical cell into a secondary oxidant generator. This can be beneficial for rapid parent-compound loss in saline or high-conductivity wastewaters, but it also creates a narrower safety margin because chlorinated intermediates, chlorate, perchlorate, bromate, and absorbable organic halogens may form. Brüninghoff *et al.*^5^ observed that 4-ethylphenol degradation in high-salinity chloride media accelerated from 30 µM L^−1^ h^−1^ in 0.6 M NaNO_3_ to 1750 µM L^−1^ h^−1^ in 0.6 M NaCl, but 64–86% of non-purgeable organic carbon remained and free chlorine reached 111 mg L^−1^ after 30 min under the high-chloride condition. Bocos *et al.*^71^ similarly showed that electrocoagulation removed bronopol parent compound rapidly, yet TOC removal after 60 min remained only 14–30% across 50–300 mA, whereas longer EAOP treatments exceeded 90% TOC removal after 480 min. The contrast is important because halogen chemistry can make a treatment appear efficient if only parent disappearance is measured.

**Fig. 2:**
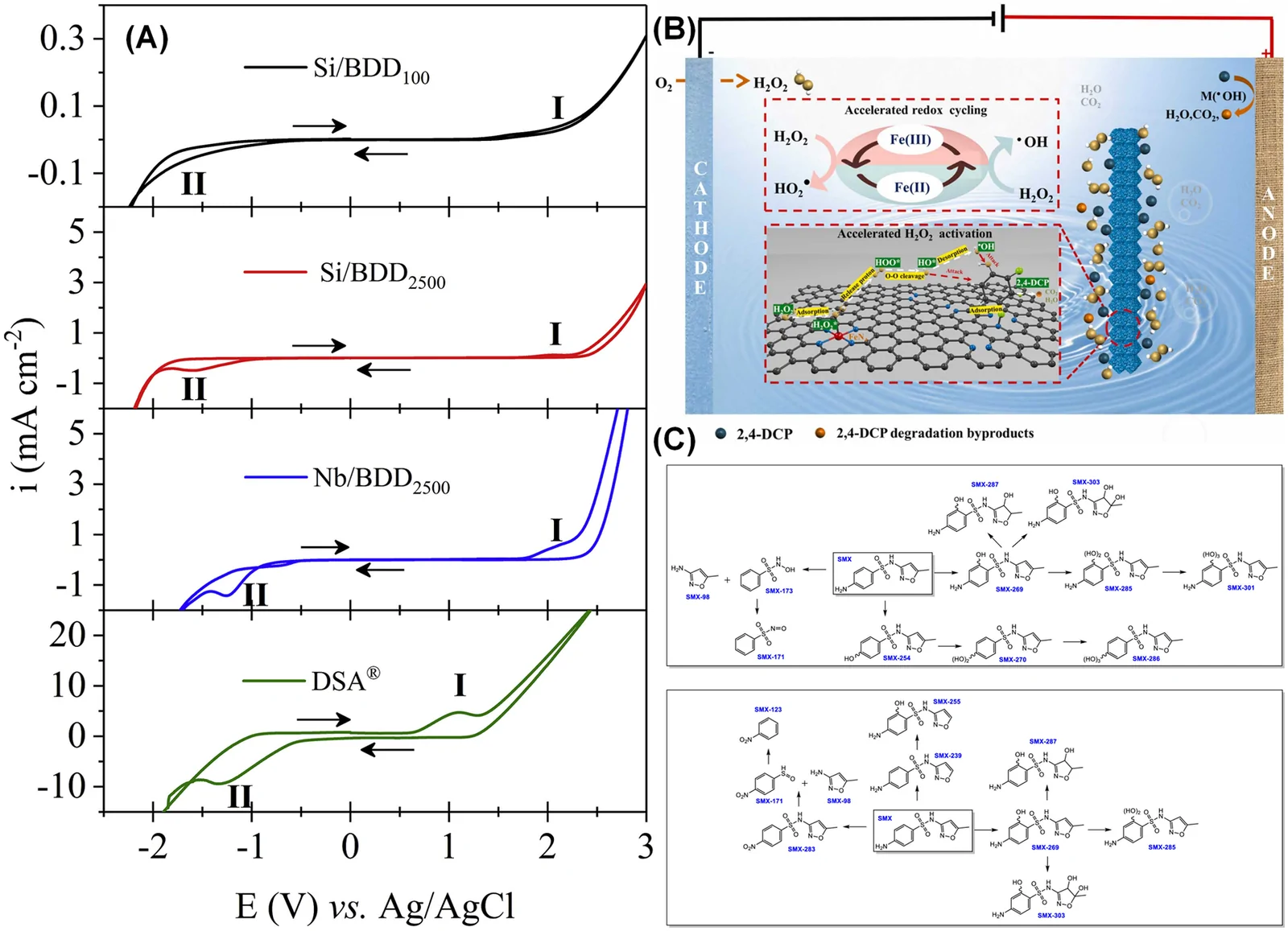
Reactive-species generation and pollutant transformation in EAOPs. (A) Anode-dependent oxidant signals.^65^. (B) H_2_O_2_ activation at Fe–N_4_ sites in a heterogeneous electro-Fenton cathode.^69^. (C) Pathway-level differences for sulfamethoxazole and hydrochlorothiazide under anodic and Fenton-type oxidation.^4^

The mechanistic evidence grouped in Fig. 2 links oxidant identification to catalytic activation and product chemistry rather than treating these as separate observations. Fig. 2(A) compares electrogenerated oxidant detection at different anodes, supporting the conclusion that anode identity controls the balance among direct oxidation, hydroxyl radicals, and secondary oxidants.^65^Fig. 2(B) represents the Fe-SAC/NC heterogeneous electro-Fenton route, where the catalyst structure explains why electrogenerated H_2_O_2_ is converted into more effective oxidative attack at Fe–N_4_ sites.^69^Fig. 2(C) maps sulfamethoxazole and hydrochlorothiazide transformation under anodic oxidation and Fenton-type routes, showing why parent-compound disappearance must be interpreted with pathway and mineralization data.^4^

### 2.3 Water-matrix effects, selectivity, and competing reactions

The water matrix determines whether the oxidant inventory generated by an EAOP is spent on the target pollutant, natural organic matter, inorganic scavengers, or secondary by-product formation. pH changes Fenton speciation, sulfate radical conversion, carbonate radical formation, and the balance between hypochlorous acid and hypochlorite. Chloride can increase apparent degradation through active chlorine, but the same pathway increases the need for by-product control. Sulfate can act as an electrolyte, a radical precursor under persulfate activation, or a spectator ion, depending on the applied process. Bicarbonate and carbonate usually scavenge hydroxyl radicals, yet carbonate-derived radicals can selectively oxidize some electron-rich structures. Nitrate, phosphate, and natural organic matter often reduce apparent kinetics by competing for oxidants and altering interfacial charge.

PFAS studies make the matrix problem especially visible because C–F bond cleavage requires strongly oxidative holes, surface-bound radicals, or specialized reductive-oxidative sequences. Wang *et al.*^72^ showed that a porous Magnéli titanium suboxide anode degraded 80.2% PFOA and 93.7% PFOS in 60 min in NaClO_4_ medium, but 20 mM nitrate lowered removal to 61.2% and 75.6%, respectively. Carbonate had the opposite effect for PFOA under the same study, increasing removal to 96.8%, while sulfate raised the rate constants from 5.86 × 10^−4^ to 6.93 × 10^−4^ s^−1^ for PFOA and from 8.09 × 10^−4^ to 1.06 × 10^−3^ s^−1^ for PFOS.^72^ In another PFAS case, Yang *et al.*^73^ found that trichloroethylene co-contamination lowered the PFOS rate constant from 0.0471 to 0.0254 min^−1^ on Ti_4_O_7_, and PFOS removal at 30 min decreased from 75% to 53%; however, fluoride release still reached about 81–83%, indicating substantial defluorination once PFOS was transformed. These two studies caution against treating anions and co-contaminants as background variables.

Real and simulated wastewaters also change the balance between parent removal and mineralization. Feng *et al.*^74^ reported complete naproxen removal within 2 h in simulated urine and wastewater during electro-Fenton treatment, while TOC removal after 4 h reached 84.6% for EF-Pt, 94.6% for EF-BDD, and 68.8% for anodic oxidation on BDD in tap-water experiments. Gadi *et al.* compared ozone, anodic oxidation, electro-Fenton, and combined EF + AO for sulfamethoxazole and hydrochlorothiazide; in real effluent, mineralization dropped from 74.29% to 47.74% for AO and from 87.9% to 84.9% for EF + AO, while ozone decreased from 23.76% to 10.9%.^4^ The combined process therefore retained mineralization better than the single processes, but it did so by increasing system complexity and pH staging. Ouarda *et al.*^75^ found that Microtox inhibition during acetaminophen-spiked secondary effluent treatment could rise before declining, which means that mineralization and bioassay response should be interpreted together rather than treated as interchangeable endpoints.

**Fig. 3:**
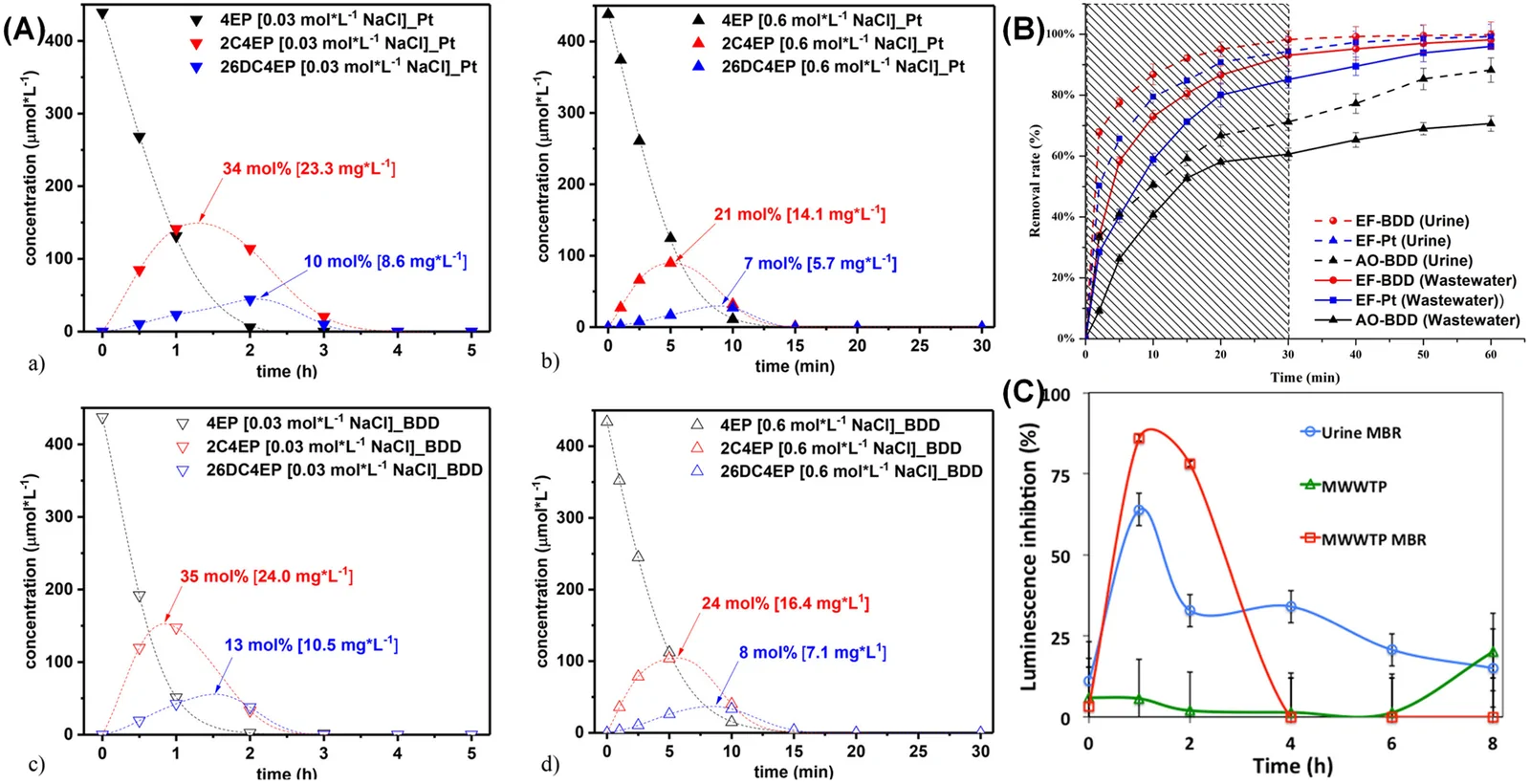
Matrix effects and toxicity-relevant products. (A) Chlorinated 4-ethylphenol intermediates in saline electrochemical treatment.^5^. (B) Naproxen removal and intermediate formation in urine and wastewater matrices.^74^. (C) Transient Microtox inhibition during acetaminophen treatment in secondary effluents.^75^

The matrix-dependent risks summarized in Fig. 3 connect directly to these quantitative trends. Fig. 3(A) shows chlorinated 4-ethylphenol products in saline electrochemical degradation, explaining why chloride can accelerate parent loss while increasing chlorinated-intermediate risk.^5^Fig. 3(B) compares naproxen removal in urine and wastewater matrices, where complete parent removal within 2 h did not remove the need to evaluate TOC and transient toxicity.^74^Fig. 3(C) tracks Microtox inhibition during acetaminophen treatment in secondary effluents and demonstrates that toxicity may increase before declining, especially when nitrogen-containing or chlorinated products persist.^75^

### 2.4 By-product formation and toxicity windows under operating control

Side reactions arise when oxidant flux exceeds the rate at which the target and its intermediates can be mineralized, or when matrix ions enter faster competing pathways. Applied potential and current density are therefore not simple intensity controls. Raising them increases interfacial oxidant production, but can also accelerate oxygen evolution, electrode corrosion, active-chlorine formation, and sequential conversion of hypochlorite to chlorate and perchlorate. In a reactive electrochemical membrane, increasing current density from 73 to 290 A m^−2^ increased perchlorate from about 130 µg L^−1^ to 25 mg L^−1^ even as throughput increased.^76^

Halide concentration determines both rate and product spectrum. Chloride can shift a cell from short-lived surface ˙OH toward longer-lived HOCl/OCl^−^, which improves transport-limited parent removal but increases chlorinated organics, AOX, chlorate, and perchlorate. Bromide-containing pollutants add brominated intermediates and bromate risk.^5,71^ At a fixed oxidant flux, increasing the initial organic concentration lowers the oxidant-to-carbon ratio and promotes partially oxidized products; malachite-green removal, for example, declined from complete removal at or below 1000 mg L^−1^ to 65.0% at 3000 mg L^−1^ under the same 30 min treatment window.^68^

pH changes both catalyst speciation and oxidant selectivity. Acidic conditions favor soluble Fe^2+^/Fe^3+^ cycling in conventional electro-Fenton chemistry, whereas near-neutral operation can precipitate iron or require heterogeneous sites. pH also controls the HOCl/OCl^−^ ratio and the conversion of SO_4_˙^−^ to ˙OH. Treatment time and oxidant dose create a toxicity window: early hydroxylation, chlorination, or ring opening can transiently increase bioassay response before further oxidation lowers it, as observed for acetaminophen and naproxen matrices.^74,75^

**Fig. 4:**
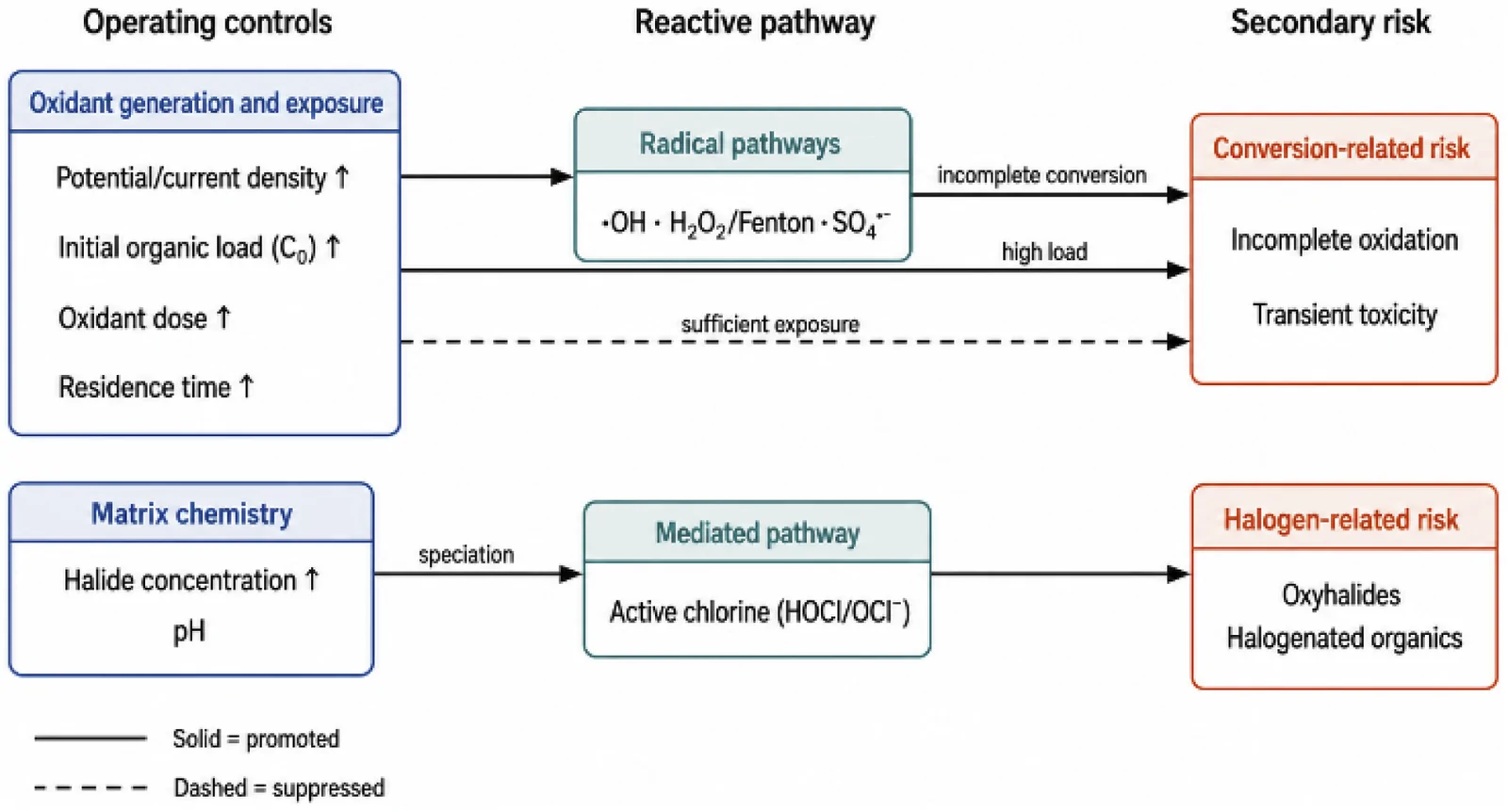
Operating-condition-to-by-product and toxicity risk map for EAOPs. Applied potential/current density, halide concentration, pH, initial organic load, oxidant dose, and residence time are connected to radical or mediated pathways, incomplete oxidation, oxyhalides, halogenated organics, and transient toxicity. Solid and dashed arrows distinguish promoted and suppressed pathways, respectively.

A defensible operating window should therefore be selected by simultaneously tracking parent concentration, TOC or COD, targeted halogen oxyanions, non-target transformation products, residual oxidants, and at least one matrix-appropriate bioassay. Fig. 4 summarizes this causal map and distinguishes conditions that increase oxidant availability from conditions that increase secondary risk.

Table 1 is organized by oxidant pathway and uses a five-column decision structure rather than a list of unrelated parameters. Every row states the initial concentration and matrix, controlling window, comparable endpoint package, and missing risk or transferability evidence. Values are retained in the units reported by the source; absent values are marked NR rather than inferred.

**Table 1:** Pathway-based evidence matrix for EAOP treatment, with initial load, operating window, endpoint package, and transferability risk^a^

Ref.	System context (*C*_0_ and matrix)	Pathway and controlling window	Comparable endpoint package	Transferability and risk
72	*C*_0_/matrix: PFOA/PFOS, 2 µM each, synthetic electrolyte with anions	Direct anodic oxidation on porous Magneli Ti suboxide; 10 mA cm^−2^; 100 mM NaClO_4_; selected anions at 20 mM	Endpoints: 60 min removal: PFOA 80.2%, PFOS 93.7%; nitrate lowered removal to 61.2% and 75.6%; carbonate raised PFOA removal to 96.8%	Decision note: matrix ions changed kinetics substantially; mineralization and energy were not reported
77	*C*_0_/matrix: PFOA/PFOS, 0.2/2 ppm, synthetic matrix	EO and EO-EF on Ti_4_O_7_ Magneli anode; 13 mA cm^−2^; 50 mM Na_2_SO_4_; 5 h; acidic EO-EF	Endpoints: EO removed 52% PFOA and 82% PFOS; EO-EF removed 92% PFOA and 100% PFOS; PFOA energy to 50% degradation fell from 14.91 to 3.95 kWh m^−3^	Decision note: electro-Fenton improved PFAS removal but required pH and Fe/O_2_ management
68	*C*_0_/matrix: malachite green, up to 3000 mg L^−1^	Electro-Fenton with iron electrodes and H_2_O_2_; pH 3; 10 mA cm^−2^; NaCl; 50 mg L^−1^ H_2_O_2_	Endpoints: complete removal within 15 min at optimum; TOC removal 95.3% after 30 min for 200 mg L^−1^; *k* = 0.092 min^−1^ for EF *vs.* 0.020 min^−1^ for EC	Decision note: high performance at acidic pH; iron sludge and high-strength dye demand remain concerns
74	*C*_0_/matrix: Naproxen, 0.198 mM, tap water/simulated urine/wastewater	Electro-Fenton and anodic oxidation on Pt/BDD with carbon felt; pH 3 for EF; 0.10–0.75 A	Endpoints: complete naproxen removal; 100% in simulated urine/wastewater within 2 h at 0.30 A; TOC after 4 h: EF-Pt 84.6%, EF-BDD 94.6%, AO-BDD 68.8%	Decision note: toxicity rose early before declining; urine/wastewater matrices complicate interpretation
73	*C*_0_/matrix: PFOS, 2 µM, with or without 76 µM trichloroethylene	Ti_4_O_7_ anodic oxidation; 10 mA cm^−2^; 100 mM Na_2_SO_4_; current density range 2.5–20 mA cm^−2^	Endpoints: PFOS removal at 30 min was 75% without TCE and 53% with TCE; k decreased from 0.0471 to 0.0254 min^−1^; fluoride release about 81–83%	Decision note: co-contaminants slowed PFOS transformation; chlorine oxyanion by-products require control
67	*C*_0_/matrix: 4-chlorophenol, 100 µM	Flow-through reactive electrochemical membrane with defective TiO_2_ on porous Ti; pH 7; 19.7 mA cm^−2^; 0.33 M NaClO_4_; 13.6–54.5 s residence	Endpoints: 7 µm membrane removed 94.0% parent in 13.6 s and 96% TOC in 54.5 s; energy for 90% removal was 0.86 kWh m^−3^	Decision note: excellent transport intensification; fouling and scale-up durability remain unresolved
70	*C*_0_/matrix: bisphenol A, 2 mg L^−1^	Electro-enhanced peroxymonosulfate activation on Fe-SAC/CF cathode; pH 3–10; 50 mM Na_2_SO_4_; PMS addition	Endpoints: cathodic *k* = 0.073 min^−1^*vs.* anodic *k* = 0.015 min^−1^; continuous removal stayed 99.5–97.5% over 17 h; EEC = 0.15 kWh log^−1^ m^−3^	Decision note: external PMS cost and sulfate-containing residuals must be included in assessment
66	*C*_0_/matrix: methyl orange, synthetic sulfate electrolyte	Mixed-metal oxide anodic oxidation; pH 7; Na_2_SO_4_; 7.1, 17.8, and 70 mA cm^−2^	Endpoints: Ti/RuO_2_ color removal 76–85%; selected mixed-oxide anodes reached 96–98%; best TOC removal 76.0%	Decision note: higher current increased voltage from 4.5 to 8.1 V; color removal exceeded mineralization
69	*C*_0_/matrix: 2,4-dichlorophenol, 0.14 mM	Heterogeneous electro-Fenton over Fe-SAC/NC with air-diffusion GDE; pH 4; 50 mA; 0.05 M Na_2_SO_4_	Endpoints: complete degradation in 90 min; EO-H_2_O_2_ alone removed 17.3%; BDD/air-diffusion HEF reached 67.6% mineralization at 300 min	Decision note: catalyst synthesis, GDE wetting, and long-term iron-site stability remain scale-up barriers
65	*C*_0_/matrix: Norfloxacin, 0.1 mM, synthetic and real wastewater	BDD/DSA anodic oxidation; 10 mA cm^−2^; 0.01 M Na_2_SO_4_	Endpoints: *k* = 0.75 h^−1^ in synthetic electrolyte, 0.82 h^−1^ in sulfate-amended real wastewater, and 0.45 h^−1^ in unamended real wastewater; Nb/BDD required about 60% less energy than Si/BDD	Decision note: conductivity and anode substrate strongly affected energy-normalized performance
71	*C*_0_/matrix: bronopol, 2.78 mM	Electrocoagulation, EO-H_2_O_2_, and electro-Fenton; 50–300 mA; BDD/Pt comparisons	Endpoints: EC removed parent rapidly but TOC after 60 min was 14–30%; EAOPs exceeded 90% TOC after 480 min; EC/EF-BDD reached 85% TOC after 420 min	Decision note: brominated intermediates and bromate risk complicate halogen-containing pollutants
5	*C*_0_/matrix: 4-ethylphenol, about 50 mg L^−1^, saline media	Electrochemical degradation and photochemical-assisted variants; Ti/Pt/BDD; 15 mA cm^−2^; NaCl or NaNO_3_	Endpoints: zero-order rate increased from 30 µM L^−1^ h^−1^ in 0.6 M NaNO_3_ to 1750 µM L^−1^ h^−1^ in 0.6 M NaCl; 64–86% organic carbon remained	Decision note: active chlorine accelerated parent loss but produced chlorinated intermediates and residual chlorine
4	*C*_0_/matrix: sulfamethoxazole and hydrochlorothiazide, 40 mg L^−1^ each	Ozone, AO, EF, and EF + AO on BDD mesh/carbon felt; 50 mM Na_2_SO_4_; EF at pH 3; AO at pH 7–8; 150–500 mA	Endpoints: in real effluent, mineralization was 10.9% for ozone, 47.74% for AO, and 84.9% for EF + AO; synthetic EF achieved 88% TOC *vs.* AO 74% and ozone 24%	Decision note: combined treatment improved mineralization but increased operating complexity

a Comparison note: *C*_0_ denotes initial concentration and NR denotes not reported. Source units are retained; missing values are not inferred. The final column identifies the endpoint that limits comparison or deployment.

Across Table 1, intensified oxidant production, high conductivity, acidic Fenton conditions, and flow-through transport often improve parent removal. The discriminating endpoints are instead carbon conversion, energy per defined treatment depth, toxicity evolution, and by-product formation. The reactive membrane combined rapid removal with 96% TOC conversion at sub-minute residence time,^67^ whereas saline 4-ethylphenol treatment accelerated parent loss but left most organic carbon and generated residual chlorine.^5^ Section 3 therefore asks how electrode and reactor design can place the system inside a useful kinetic window without shifting the burden to energy, durability, or secondary pollution.

## 3. Electrode materials, reactor architectures, and operating windows

Section 3 translates the chemistry established in Section 2 into engineering choices. It proceeds from anode selectivity and stability, to cathodic H_2_O_2_ production and paired reactions, and finally to hydrodynamic architectures that control mass transfer, residence time, and auxiliary energy.

### 3.1 Anodes: BDD, PbO_2_, SnO_2_–Sb, Ti_4_O_7_, MMO/DSA, and carbon-based electrodes

Electrode selection determines the upper limit of EAOP performance, but the apparent ranking of anodes changes when parent-compound removal, mineralization, energy, and by-product formation are evaluated together. BDD remains the benchmark for non-active anodic oxidation because it can generate weakly adsorbed hydroxyl radicals with high oxygen-evolution overpotential. In a divided-cell nitrofurazone study, BDD destroyed nearly all of the parent compound within 30 min at 0.1 A cm^−2^ and reduced TOC to below 1 mg L^−1^, whereas platinized titanium achieved about 95% parent removal but left higher COD and TOC residues.^78^ The BDD first-order constant was 0.16 min^−1^ and was 2–2.5 times that of Pt/Ti under the extracted conditions.^78^ This comparison supports the strong-oxidant value of BDD, but it also shows a recurring limitation: chloride-mediated routes can form chlorinated intermediates, so high parent removal is not enough for safety assessment.

PbO_2_- and SnO_2_-based anodes can be less expensive or easier to coat on Ti substrates, but they introduce stability and metal-release questions that require explicit reporting. Weng *et al.*^79^ showed that Ce-doped PbO_2_ removed 96.96% of 500 mg L^−1^*para*-aminophenol in 180 min, compared with 89.34% for La–PbO_2_ and 77.55% for undoped PbO_2_; COD removal followed the same order at 73.79%, 68.49%, and 59.49%, and the final electric energy per order decreased from 51.79 kWh m^−3^ for PbO_2_ to 22.22 kWh m^−3^ for Ce–PbO_2_. The improvement is meaningful, but the review value of such data depends on whether Pb leaching, coating lifetime, and end-of-life disposal are quantified. For cefuroxime, a Sb–SnO_2_–Ni/Ti mesh anode achieved complete parent removal and COD/TOC depletion within 60 min at pH 7, 50 mA cm^−2^, and 750 mg L^−1^ KCl, but anodes failed at 75–100 mA cm^−2^ and the process relied on active chlorine chemistry.^80^ These examples show that doped oxide anodes should be discussed as application-specific materials rather than as universal replacements for BDD.

Mixed-metal oxides and Pt-based anodes often perform well for mediated oxidation, but they are more sensitive to the chosen oxidant pathway. In pulsed anodic oxidation of 50 ppm alachlor on Pt, the best power-pulse mode reached complete parent degradation after 180 min, but mineralization after 240 min was only 38.3%.^81^ The result is important because it decouples parent removal from carbon conversion even under an optimized electrical waveform. For caffeine degradation on BDD, Degermenci *et al.*^82^ reported 98.5% removal after 45 min at pH 3 and 20 mA cm^−2^; at natural pH with 50 mM K_2_SO_4_, removal after 120 min rose from 75.2% to 96.3% as current density increased from 5 to 20 mA cm^−2^, but energy increased from 3.80 to 18.75 kWh m^−3^. A material choice is therefore incomplete without a current-density window.

**Fig. 5:**
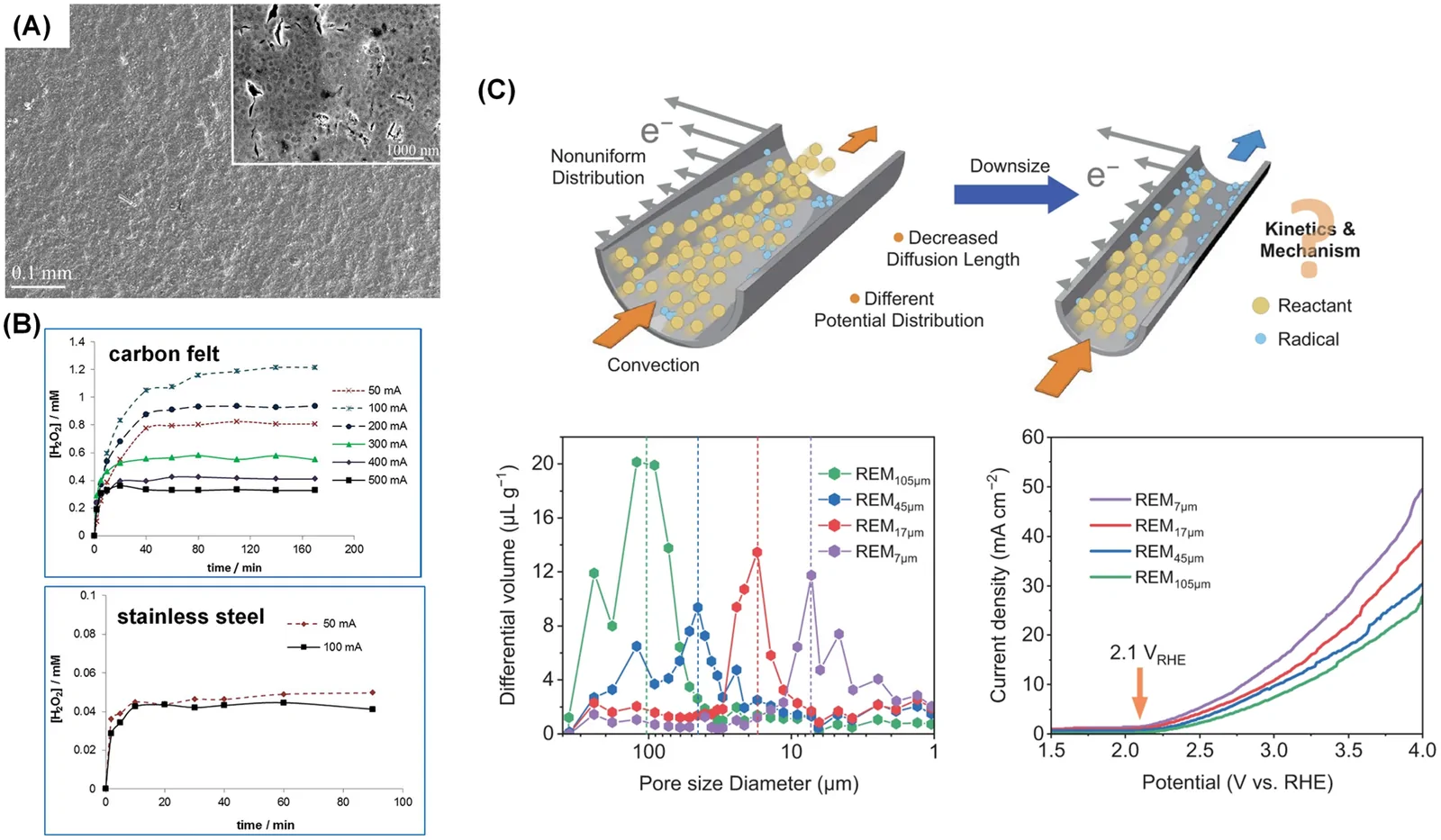
Electrode, catalyst, and membrane evidence. (A) Surface morphology of a mixed-oxide Ti/Ir–Pb anode.^66^. (B) Cathode-dependent H_2_O_2_ generation in electro-Fenton cells.^83^. (C) Pore-scale architecture and electrochemical response of reactive membranes.^67^

Material selection requires evidence that spans morphology, oxidant-generation function, and reactor structure, as represented in Fig. 4. Fig. 4(A) shows the FE-SEM morphology of a Ti/Ir–Pb anode, linking coating structure to the mixed-oxide behavior discussed for methyl orange oxidation.^66^Fig. 4(B) compares cathode-dependent H_2_O_2_ generation in electro-Fenton cells, supporting the quantitative cathode effects later summarized for carbon sponges and carbon felt.^83^Fig. 4(C) presents the structure and electrochemical properties of a reactive electrochemical membrane, connecting nanoscale electrode architecture to the residence-time and energy benefits of flow-through treatment (Fig. 5).^67^

### 3.2 Cathodes and paired oxidation-reduction designs

Cathode design is the limiting component in many electro-Fenton and paired oxidation-reduction systems. The cathode must reduce oxygen to H_2_O_2_ at high current efficiency while suppressing hydrogen evolution, H_2_O_2_ decomposition, and mass-transfer loss. Sopaj *et al.*^83^ compared carbon sponges with different pore densities, carbon felt, and stainless steel cathodes for sulfamethazine degradation at pH 3 with a Pt anode and Fe^2+^ catalyst. At 300 mA, TOC removal after 8 h was 91.1% and 91.2% for 45 and 60 ppi carbon sponges, but only 55.6% for carbon felt and 37.2% for stainless steel; the corresponding apparent degradation constants at 300 mA were 0.60, 0.50, 0.16, and 0.07 min^−1^, respectively.^83^ The same study reported that the 45 ppi sponge generated 3.5 mM H_2_O_2_ at 100 mA, 2.6 times the maximum obtained with carbon felt.^83^ This quantitative spread is larger than many anode-comparison studies and shows why cathodes should not be treated as passive counter electrodes.

Gas-diffusion, carbon-felt, sponge, and pressurized cathode designs represent different ways to improve oxygen availability. Scialdone *et al.*^84^ used a compact high-pressure electro-Fenton cell and showed that raising air pressure from 1 to 6 and 11 bar increased Acid Orange 7 TOC abatement from 48% to 63% and about 74% after 7 h at 110 mA. The authors attributed the gain to enhanced H_2_O_2_ generation and reduced hydrogen evolution, while also estimating that compression cost was lower than the electrochemical energy saved.^84^ This is a valuable scale-up concept because it improves cathode performance without relying only on larger electrode area, yet the small high-pressure cell also introduces pressure hardware and safety requirements that rarely appear in beaker-scale comparisons.

Paired designs become most convincing when they exploit both electrodes rather than using one electrode merely to close the circuit. Three-dimensional electrochemical cells, electro-Fenton/anodic oxidation combinations, and sequential electrocoagulation-electrooxidation systems can remove different fractions of the contaminant load at different stages. In a 3D methylene blue cell, fly ash-red mud particle electrodes gave 86.46% dye removal at pH 7 and 9 V, compared with 56.49% for electrocoagulation, and the electrochemical oxidation energy consumption was 21.35 kWh m^−3^ compared with 81.51 and 36.55 kWh m^−3^ for electro-Fenton and electrocoagulation comparators in the extracted row.^85^ The improvement is promising, but the absence of TOC data prevents a mineralization claim. This distinction will recur throughout the table.

### 3.3 Flow-by, flow-through, 3D particle, membrane-assisted, and gas-diffusion reactors

Reactor architecture translates electrode chemistry into treatment capacity. In flow-by reactors, pollutants reach the anode through diffusion and convection across a boundary layer. In flow-through reactors and reactive electrochemical membranes, the water is forced through pores or conductive media, shortening the mass-transfer distance and increasing the probability that oxidants react before they decay. Three-dimensional particle-electrode systems add a packed or suspended conductive phase, increasing apparent surface area and enabling adsorption-electrooxidation coupling. These gains are not free: particle attrition, short-circuiting, pressure drop, cathode wetting, gas management, and fouling become central operational variables.

The 3D electrochemical literature shows both the value and ambiguity of added particulate electrodes. Correia-Sá *et al.*^86^ reported that 3D treatment with biochar or activated carbon achieved 95% carbamazepine removal after 30 min, while a 2D process reached 80% at the same time and 87% at 45 min. The energy indicators were also lower for the best biochar condition, with 390 Wh g^−1^ and 2709 kWh m^−3^ per order compared with 418 Wh g^−1^ and 7507 kWh m^−3^ for 2D treatment.^86^ However, the same row lacks TOC and transformation-product evidence, and the process combines adsorption, electrosorption, and electrooxidation. For diclofenac and sulfamethoxazole, Soares *et al.*^87^ showed the matrix penalty directly: complete removal in clean aqueous solution did not transfer to fortified WWTP effluent, where 30 min treatment removed 49% diclofenac and 86% sulfamethoxazole, with energy demands of 1224 and 613 Wh g^−1^, respectively. Particle electrodes can therefore improve kinetics, but they do not remove the need for real-matrix validation.

Filter-press, continuous-flow, and pre-pilot systems provide stronger evidence for translation. Coledam *et al.*^88^ treated 100 mg L^−1^ norfloxacin in a filter-press BDD reactor and obtained complete parent removal at 10 mA cm^−2^, with about 90% organic-load removal after 5 h and first-order constants of about 2 × 10^−2^ min^−1^ for norfloxacin and 6 × 10^−3^ min^−1^ for TOC. Detoxification lagged behind parent removal, as *E. coli* inhibition became almost null only after about 4 h.^88^ Gökkuş *et al.*^89^ used continuous-flow electrocoagulation followed by electro-Fenton or photoelectro-Fenton for Acid Brown 14 and obtained complete color loss during EC and up to 96.7% TOC removal after the EC-PEF train in sulfate medium. The energetic comparison was less favorable: EC required 10.41–12.47 kWh m^−3^, EC-EF required 79.57–86.27 kWh m^−3^, and EC-PEF required about 470 kWh m^−3^ when the UVA lamp was included.^89^ Thus, intensification can improve mineralization but may shift the limiting factor from chemistry to electrical or photon energy.

**Fig. 6:**
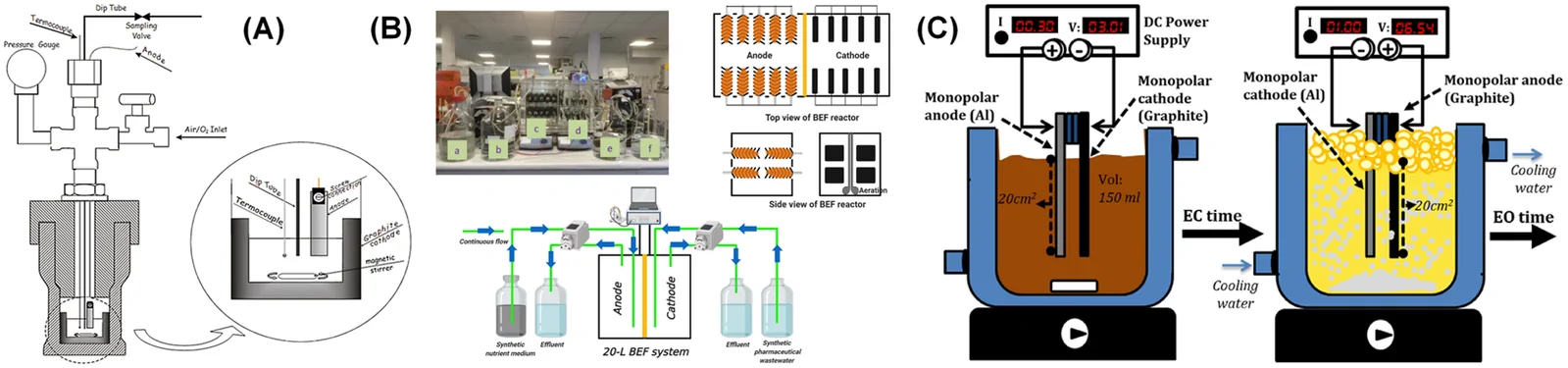
Reactor configurations relevant to scale-up. (A) Pressurized electro-Fenton cell for enhanced oxygen transfer and H_2_O_2_ generation.^84^. (B) 20 L continuous-flow bio-electro-Fenton reactor.^90^. (C) Staged electrocoagulation-electrooxidation train.^93^

Reactor-scale evidence determines whether electrode performance can be translated beyond batch screening. Fig. 6(A) shows a pressurized electro-Fenton cell that intensifies oxygen transfer,^84^ while Fig. 6(B) shows a 20 L continuous-flow bio-electro-Fenton system in which residence time and auxiliary energy become explicit design variables.^90^ Water falling-film reactors provide a complementary route to intensification. A thin gravity-driven film increases gas–liquid, photon–liquid, or plasma–liquid interfacial area and shortens diffusion distances. Using a common planar falling-film design, ozonation, photocatalytic ozonation, photocatalysis, and dielectric-barrier-discharge plasma were compared for diclofenac and ibuprofen; the study showed that high degradation energy yield did not guarantee mineralization.^91^ The broader falling-film literature identifies film thickness, wetting uniformity, recirculation, photon or gas distribution, surface fouling, and scale-dependent hydrodynamics as the variables that must be reported before performance can be generalized.^92^Fig. 6(C) completes the reactor sequence with an integrated electrocoagulation-electrooxidation system, where each stage has a distinct solids-removal or oxidation role.^93^

Table 2 applies the same five-column structure to materials and reactors. Electrode or reactor identity is combined with target and matrix context; the controlling window is separated from the endpoint package; and the final column states the principal translation risk.

**Table 2:** Design-based evidence matrix for electrodes and reactor architectures, with normalized operating windows and explicit missing endpoints^a^

Ref.	Design and matrix context	Normalized operating window	Comparable endpoint package	Transferability and risk
85	3D EO with iron anode, graphite cathode, and fly ash-red mud particles; *C*_0_/matrix: Methylene blue, 10 mg L^−1^; synthetic solution and surface-water validation	pH 7; 9 V; 0.1 M Na_2_SO_4_; 100 g L^−1^ particles	Endpoints: 86.46% MB removal; EC comparator 56.49%; EO energy 21.35 kWh m^−3^*vs.* 81.51 for EF and 36.55 for EC	Decision note: particle electrodes improved removal and energy, but TOC was not reported and real surface water lowered removal by 18.84%
78	BDD *vs.* Pt/Ti in divided cell; *C*_0_/matrix: Nitrofurazone, 10–100 mg L^−1^; chloride-containing model solution	pH about 2; 0.1 A cm^−2^; 30 min	Endpoints: BDD gave about 100% destruction and TOC below 1 mg L^−1^; Pt/Ti gave about 95% destruction; BDD *k* = 0.16 min^−1^	Decision note: strong BDD oxidation, but chloride-mediated intermediates require by-product monitoring
79	Ce–PbO_2_, La–PbO_2_, and PbO_2_ anodes; *C*_0_/matrix: *para*-aminophenol, 500 mg L^−1^; synthetic sulfate matrix	70 mA cm^−2^; 180 min; 0.1 M Na_2_SO_4_	Endpoints: PAP removal 96.96%, 89.34%, and 77.55%; COD removal 73.79%, 68.49%, and 59.49%; EE/O 22.22, 34.56, and 51.79 kWh m^−3^	Decision note: Ce doping improved kinetics and energy, but lead-based anode safety was not resolved
82	BDD with cathode comparison; *C*_0_/matrix: Caffeine, 25–75 mg L^−1^; K_2_SO_4_ model solution	5–20 mA cm^−2^; optimum pH 3; 45–120 min	Endpoints: 98.5% removal after 45 min at pH 3; at natural pH, removal increased from 75.2% to 96.3% as current density rose from 5 to 20 mA cm^−2^; energy rose from 3.80 to 18.75 kWh m^−3^	Decision note: higher current improved removal but increased energy; no TOC or by-product endpoint
86	3D carbon particulate process with MMO anode; *C*_0_/matrix: carbamazepine, 10 mg L^−1^; ultrapure-water NaCl matrix	pH 7; 6.67 mA cm^−2^; 0.150 g particulate carbon; 30 min	Endpoints: 3D process reached 95% removal after 30 min; 2D reached 80%; best biochar half-life 5.8 min; EEO 2709 *vs.* 7507 kWh m^−3^ for 2D	Decision note: mixed adsorption/electrooxidation mechanism; no mineralization or by-product data
94	Graphene/metal-oxide composite electrodes on stainless steel; *C*_0_/matrix: Phenolic dye mixture, 15 ppm; Na_2_SO_4_ model solution	10 mA cm^−2^; 6 h	Endpoints: about 86% degradation and 30–35% TOC mineralization; peak-current change below 10% over ten cycles	Decision note: low-cost electrodes were stable, but treatment was slow and mineralization partial
80	Sb–SnO_2_–Ni/Ti mesh anode; *C*_0_/matrix: cefuroxime, 50 mg L^−1^; synthetic solution with KCl	pH 7; 50 mA cm^−2^; 750 mg L^−1^ KCl; 60 min	Endpoints: complete parent, COD, and TOC removal after 60 min; high current densities of 75–100 mA cm^−2^ broke the anodes	Decision note: effective but strongly dependent on chloride-mediated oxidation and anode stability
95	Low-cost graphite/copper electrode pairs; C_0_/matrix: paracetamol and propranolol, 15 mg L^−1^ each; aqueous and synthetic effluent	18 V for paracetamol; 5 V for propranolol; 75–150 min	Endpoints: aqueous paracetamol complete after 75 min; synthetic effluent after 150 min: Paracetamol 84.53%, propranolol 52.78–62.95%	Decision note: UV/Vis removal did not prove mineralization; seed assays showed toxicity after treatment
87	3D biochar particle electrode with MMO anode; *C*_0_/matrix: Diclofenac and sulfamethoxazole, 10 mg L^−1^; aqueous and WWTP effluent	pH 7; 7 mA cm^−2^; 30 min	Endpoints: clean-water removal complete; fortified WWTP effluent removal was 49% DCF and 86% SMX; EE/O 19.1 and 8.77 kWh m^−3^	Decision note: matrix competition sharply reduced removal; mineralization and chlorinated products were not reported
81	Pulsed Pt anodic oxidation; *C*_0_/matrix: Alachlor, 50 ppm; tap water with sulfate	Natural pH 7.8; power pulse up to 2.5 W; 180–240 min	Endpoints: best pulse mode achieved 100% degradation after 180 min; mineralization was 38.3% after 240 min	Decision note: pulse operation improved parent degradation but did not deliver deep mineralization
83	Carbon sponge cathodes for electro-Fenton; *C*_0_/matrix: Sulfamethazine, about 0.1 mM; sulfate model solution	pH 3; 50–500 mA; Fe^2+^ 0.2 mM; 8 h	Endpoints: at 300 mA, TOC removal was 91.1% for 45 ppi sponge *vs.* 55.6% for carbon felt and 37.2% for stainless steel; *k* = 0.60, 0.16, and 0.07 min^−1^	Decision note: cathode pore structure controlled H_2_O_2_ generation and mineralization efficiency
89	Continuous EC followed by EF or PEF; *C*_0_/matrix: Acid Brown 14, 50 mg L^−1^ TOC; chloride or sulfate media	EC 18 min; EF/PEF 540 min; *j* = 50 mA cm^−2^ for EF/PEF	Endpoints: EC removed 28.9–34.9% TOC; EC-EF about 68%; EC-PEF up to 96.7%; total PEF energy about 470 kWh m^−3^	Decision note: mineralization improved, but UVA lamp energy dominated process cost
88	BDD filter-press flow reactor; *C*_0_/matrix: Norfloxacin, 100 mg L^−1^; sulfate model solution	10 mA cm^−2^; flow 420 L h^−1^; 300 min	Endpoints: complete parent removal and about 90% organic-load removal after 5 h; *k*_NOR about 2 × 10^−2^ min^−1^ and *k*_TOC about 6 × 10^−3^ min^−1^	Decision note: mass-transfer-limited flow operation; detoxification required much longer than parent removal
84	Pressurized electro-Fenton cell; *C*_0_/matrix: Acid orange 7, 0.43 mM; sulfate model solution	pH 3; 110 mA; air pressure 1–11 bar; 7 h	Endpoints: increasing pressure from 1 to 6 and 11 bar raised TOC abatement from 48% to 63% and about 74%	Decision note: pressure improved H_2_O_2_ generation but adds hardware and safety constraints

a Comparison note: *C*_0_ denotes initial concentration and NR denotes not reported. Source units are retained; missing values are not inferred. The final column identifies the endpoint that limits comparison or deployment.

Table 2 shows that robust designs report a carbon endpoint and an operational endpoint in addition to parent removal. BDD and doped oxides can be effective, but their advantage shrinks when energy, metal release, active-chlorine products, or detoxification time are included. Three-dimensional and flow-through reactors shorten transport distances, yet adsorption and oxidation must be separated experimentally. Cathode pore geometry and oxygen delivery also change H_2_O_2_ production by several fold. Once these material and transport windows are defined, Section 4 asks how pollutant structure changes the endpoint needed to claim treatment success.

## 4. Pollutant classes, degradation performance, and transformation risk

Section 4 applies the coupled framework to pollutant classes. It first relates molecular structure to reactivity, then separates degradation from mineralization and detoxification, and finally derives class-specific rules for energy and by-product assessment.

### 4.1 Pharmaceuticals, endocrine disruptors, dyes, phenols, pesticides, PFAS, and industrial additives

Persistent organic pollutants do not form a single electrochemical class. Their behaviour depends on functional groups, ionization state, adsorption affinity, bond dissociation energy, and whether transformation produces more or less persistent fragments. Pharmaceuticals and endocrine-disrupting compounds often contain electron-rich aromatic rings, amine groups, heterocycles, sulfonamides, or carboxylates that react with hydroxyl radicals and active chlorine, but their partial oxidation can produce intermediates with distinct toxicity. Dyes and phenols may decolorize rapidly because chromophores are attacked before the carbon skeleton is fully mineralized. PFAS are a separate challenge because the C–F bond and perfluoroalkyl chain require interfacial electron-transfer or highly oxidative conditions rather than conventional hydroxyl-radical chemistry alone. Industrial additives, nitroaromatics, pesticides, and surfactants add further complexity because their matrices often include salts, solvents, suspended solids, and co-contaminants.

Pharmaceuticals provide the clearest example of structure-dependent performance. Parabens are relatively reactive toward hydroxyl radicals and photoelectro-Fenton chemistry, but their alkyl-chain length and concentration still influence removal. In a solar photoelectro-Fenton study of methyl-, ethyl-, and propylparaben in real secondary effluent, BDD-based treatment removed all three parabens within 150 min at 10 mA cm^−2^, whereas a RuO_2_-based anode required 180 min for greater than 95% removal.^96^ At a more realistic 30 µM concentration, methylparaben still exceeded 95% removal after 180 min, but ethylparaben and propylparaben declined to 88% and 60%, respectively.^96^ The same study reported 66% TOC abatement for BDD and 47–52% for the RuO_2_-based configuration in real wastewater, indicating that trace-level kinetics and carbon conversion should not be inferred from high-concentration parent removal.^96^

Antibiotics span several mechanistic regimes. Sulfonamides, fluoroquinolones, tetracyclines, and nitrofurans contain heteroatoms and aromatic systems that can be attacked by radicals, but they may retain antimicrobial or ecotoxic activity until key pharmacophores are broken. A bio-electro-Fenton system degraded 99.04% of tetracycline after 24 h and removed 85.71% of anodic COD under the optimized substrate condition, while also generating bioelectricity.^97^ This shows that hybrid bioelectrochemical routes can reduce external energy demand, but the 24 h reaction time, pH 3 catholyte, and FeSO_4_ dosing make it unsuitable as a direct substitute for high-rate anodic oxidation in all applications.^97^ Fluoroquinolone reviews emphasize persistence, ecotoxicity, and resistance-selection concerns, which justify monitoring beyond disappearance of the parent antibiotic.^98^

Dyes and phenolic compounds are often used as model pollutants because UV-vis decolorization is convenient, but that convenience can be misleading. In phenolic dye mixtures, low-cost graphene/metal-oxide composite electrodes produced about 86% degradation after 6 h but only 30–35% TOC mineralization.^94^ In active-chlorine-mediated azo-dye oxidation, Fenton-like reactions with active chlorine can improve apparent degradation, but the same chemistry creates a need to identify chlorinated intermediates rather than relying on color loss.^99^ Phenols are also prone to coupling, hydroxylation, ring opening, and short-chain acid formation. Recent work on biochar-coactivated phenols and peroxydisulfate showed that electron utilization can promote polyphenol accumulation rather than mineralization, a principle that is relevant to EAOP interpretation whenever partial oxidation products remain in solution.^100^

PFAS performance should be evaluated separately from other pollutant classes. For PFOA and PFOS, the most relevant endpoints include parent loss, chain-shortening products, fluoride release, total oxidizable precursor behaviour, and energy normalized to treatment depth. Fig. 6(B) reinforces this point because PFAS degradation can look successful by parent removal while still requiring defluorination and short-chain product accounting.^77^ Pilot-scale PFAS electrooxidation studies have begun to address concentrated waters and fractionated foam, but field translation still depends on whether energy demand, co-contaminant load, inorganic background, and short-chain PFAS formation are reported together.^101^

### 4.2 Mineralization, toxicity attenuation, and incomplete oxidation

The main cross-class lesson is that degradation, mineralization, and detoxification are different endpoints. Parent-compound disappearance measures loss of the selected analytical signal. TOC or COD reduction measures carbon conversion but does not specify product identity. Toxicity assays integrate biological response, but they depend on organism, exposure time, pH, salinity, residual oxidants, and sample quenching. Defluorination, dechlorination, nitrate/nitrite release, bromate formation, chlorate and perchlorate formation, and AOX are class-specific indicators that often decide whether an apparently effective EAOP is environmentally acceptable.

Several examples show why these endpoints must be paired. In low-cost graphite/copper anodic oxidation of paracetamol and propranolol, aqueous paracetamol disappeared completely after 75 min, but synthetic-effluent removal after 150 min reached 84.53% for paracetamol and only 52.78–62.95% for propranolol; seed toxicity tests still showed toxic potential after treatment.^95^ In acetaminophen-spiked secondary effluents, electrooxidation removed the parent compound rapidly, but toxicity control required much higher treatment energy than parent removal, and urine-derived effluent retained measurable inhibition because of nitrogen-containing and chlorinated products.^75^ In norfloxacin mineralization on BDD, antibacterial inhibition persisted after early parent disappearance and approached zero only after longer oxidation broke the fluoroquinolone structure.^88^ These findings support a conservative reporting rule: a degradation study should not imply risk reduction unless carbon removal, transformation products, and at least one toxicity or class-specific endpoint support that interpretation.

Incomplete oxidation can also create misleading improvements in biodegradability. Increasing BOD/COD can be beneficial when EAOPs are used as pretreatment before biological treatment, but it may also reflect formation of smaller oxygenated intermediates that still require downstream polishing. Electrocoagulation, adsorption, and particle-electrode systems can lower apparent pollutant concentration without oxidizing the carbon skeleton. For this reason, hybrid systems should report stage-specific mass balances. An adsorption-rich first stage may be valuable, but it must be distinguished from mineralization if the spent sorbent, sludge, or concentrated brine becomes the final waste stream.

### 4.3 Cross-pollutant patterns in rate, energy demand, and by-product formation

Across pollutant classes, three patterns are consistent enough to guide EAOP selection. First, electron-rich and chromophoric compounds often show fast parent removal, but their mineralization is slower and more energy intensive. This is common for dyes, phenols, and many pharmaceuticals. Second, halogenated or halogen-generating systems require a separate by-product strategy. Chloride can accelerate oxidation by producing active chlorine, yet by-product-control reviews emphasize that chlorate, perchlorate, AOX, and halogenated organics must be managed as process outputs rather than treated as secondary details.^2^ Studies of chlorine-mediated electrooxidation in urine further show that active and non-active anodes can differ in chlorination by-product profiles, making anode choice a by-product-control decision as much as a degradation decision.^102^ Third, persistent fluorinated compounds require defluorination or total fluorine accounting because parent loss can represent conversion to shorter-chain PFAS rather than complete destruction.

Energy demand follows these same patterns. Processes that achieve rapid parent loss through active chlorine, adsorption-assisted oxidation, or high current density may look efficient over short treatment times. Deep mineralization, detoxification, or PFAS defluorination generally needs longer operation, higher oxidant flux, or a polishing stage. This does not mean that EAOPs should always aim for complete mineralization in a single reactor. For industrial effluents, a more rational target may be partial oxidation to improve biodegradability, followed by biological or adsorptive polishing. For trace pharmaceuticals in secondary effluent, a lower-dose EAOP may be justified only if toxicity and by-products are controlled. For PFAS concentrates, electrooxidation is more defensible as a destructive treatment of a smaller stream than as a high-volume polishing step.

**Fig. 7:**
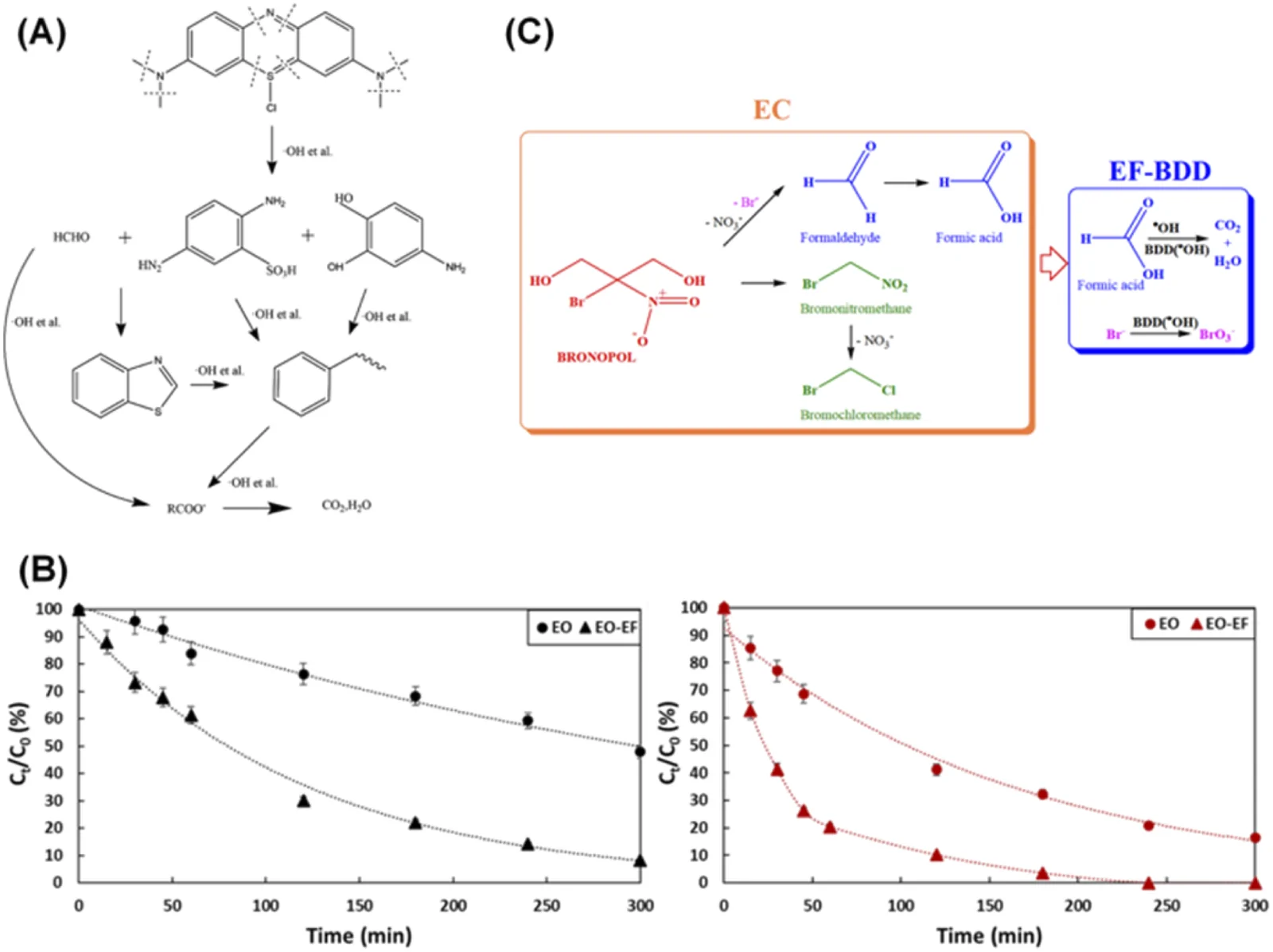
Pollutant-class-specific degradation and transformation endpoints. (A) Methylene-blue transformation pathway.^87^. (B) PFOA and PFOS degradation during coupled treatment.^77^. (C) Bronopol mineralization sequence.^71^

The cross-pollutant endpoint problem is clearest when the three classes in Fig. 7 are read together. Fig. 7(A) uses a methylene-blue pathway to show why decolorization must be interpreted through intermediate formation.^87^Fig. 7(B) compares PFOA and PFOS during electro-oxidation and electro-Fenton coupling, requiring chain-shortening and defluorination evidence.^77^Fig. 7(C) maps bronopol mineralization after sequential treatment, linking halogenated-product control to the gap between parent loss and TOC removal.^71^

A pollutant-class framework should therefore precede process optimization. Pharmaceuticals and endocrine disruptors require transformation-product and bioassay evidence; dyes and phenols require carbon conversion beyond decolorization; pesticides and nitroaromatics require ring-opening and nitrogen-species tracking; halogenated compounds require AOX and oxyhalides; and PFAS require fluoride release, short-chain products, and fluorine balance. These class-specific endpoints become the deployment criteria used in Section 5, where authentic wastewater and treatment-train roles determine whether the laboratory window remains credible.

## 5. Real wastewater applications and hybrid treatment trains

### 5.1 Industrial, municipal, landfill leachate, hospital, textile, pharmaceutical, and saline wastewaters

Real wastewater studies are essential because EAOPs respond strongly to conductivity, chloride, alkalinity, suspended solids, natural organic matter, ammonia, phosphate, and the ratio between target micropollutants and background carbon. A system that performs well in sulfate electrolyte may behave differently in textile effluent, landfill leachate, tannery wastewater, petrochemical wastewater, or municipal secondary effluent. The real-matrix literature also changes the meaning of success. In industrial wastewater, COD, TOC, color, biodegradability, and toxicity may be more relevant than removal of one spiked compound. In secondary effluent, trace pollutant removal must be weighed against dissolved organic matter oxidation and by-product formation. In PFAS concentrates, treatment is more defensible when the water stream is already concentrated by foam fractionation, adsorption, membranes, or industrial source control.

Textile wastewater has become a useful test bed because it combines color, salinity, surfactants, dye mixtures, metals, and variable biodegradability. Afanga *et al.*^103^ treated real textile effluent with sequential electrocoagulation-electro-Fenton and reduced COD from 325 to 12 mg L^−1^, corresponding to 97% COD removal, while TOC mineralization reached 97–98% and TSS removal reached 98%. The energy requirement was 0.45 kWh kg^−1^ removed TOC for the combined process, compared with 3 kWh kg^−1^ removed TOC for electrocoagulation alone.^103^ In a more saline and alkaline textile matrix, Hien *et al.*^104^ compared AO/BDD, EF/Pt, and EF/BDD and found that EF/BDD achieved 95% TOC removal after 6 h and 78% TOC removal after 4 h, with 18 kWh m^−3^ and 55 kWh kg_TOC_^−1^ at 200 mA. The same study warned that BDD operation in chloride-rich wastewater generated chlorate and perchlorate, while EF/Pt suppressed perchlorate but gave lower mineralization.^104^ These results show why real textile treatment is not just a color-removal problem.

Landfill, tannery, and petrochemical wastewaters demonstrate the other side of real-matrix operation: high organic load can be lowered substantially, but residual toxicity or energy demand may remain. A sequential membrane bioreactor plus BDD electrooxidation process removed 84.2% COD in summer landfill leachate and 56.6% in winter leachate, while TOC removal was 71.6% and 37.1%, respectively.^105^ DEHP removal reached 100% in summer and 70.6% in winter, but Daphnia toxicity units increased from 1.2 to 7.7 after the electrooxidation step, showing that advanced oxidation can increase acute toxicity when organochlorinated products or residual oxidants remain.^105^ For tannery wastewater, continuous-flow Ti/BDD electrooxidation achieved 98.45% COD removal after 6 h at 80 mA cm^−2^, whereas Ti/RuO_2_ and Ti/IrO_2_ reached 70.15% and 62.01% under the same current density.^106^ Petrochemical wastewater treated on Ru–Ir mixed metal oxide electrodes showed 32.2–79.1% COD removal within 6 min across four real samples, but energy consumption ranged from 85 to 235 kWh m^−3^ and the study did not report toxicity or halogenated by-products.^107^

### 5.2 Coupling with adsorption, membranes, biological treatment, electro-Fenton, UV, ultrasound, and persulfate activation

Hybrid treatment trains are most useful when each stage has a distinct role. Electrocoagulation can remove suspended solids, color, metals, and hydrophobic fractions before an EAOP mineralizes soluble organics. Biological treatment can reduce biodegradable COD before electrooxidation targets persistent residues. Adsorption or membranes can concentrate contaminants and reduce the volume sent to destructive treatment. UV, sunlight, ultrasound, persulfate, and peroxymonosulfate can accelerate oxidant generation, but they also add energy, reagent cost, and additional residuals.Recent adsorption and catalyst reviews sharpen the role of hybrid stages. Modified biochar can act as a low-cost polishing or concentration medium for dye-bearing streams,^108^ multifunctional MOFs can combine adsorption with catalytic degradation when mass transfer and regeneration are verified,^109^ and biochar-based catalysts can activate peroxide or persulfate for destructive oxidation.^110^ These materials are included here as complementary unit operations or catalytic supports, not as evidence that adsorption alone constitutes EAOP mineralization.

Sequential textile and coffee-effluent studies illustrate the value of staged treatment. In denim wastewater, electrocoagulation alone removed 94% of dye, while EC + EO achieved 100% color removal, 88% COD reduction, and 79% TOC removal; however, *Artemia salina* mortality rose to 100% after EO and fell to 0% only after activated-carbon polishing.^111^ This is a strong argument for hybridization because the post-polishing stage addressed a biological endpoint that electrochemical removal did not. In soluble coffee effluent, an optimized EC-EO train reduced COD by 73.90% in the verification data, removed 63.5% TOC, increased BOD_5_/COD from 0.35 to about 0.6, and saved about 34% energy and 25% operating cost relative to EO alone.^93^ The trade-off was high added NaCl and unquantified halogenated-product risk.^93^

Bioelectrochemical and membrane-assisted systems address scale-up from a different direction. A 20 L continuous-flow bio-electro-Fenton reactor completely removed six pharmaceuticals in synthetic wastewater at 0.1 V, 0.3 mM Fe^2+^, and 26 h HRT, but real secondary effluent at the same HRT removed only 37–90%; raising the applied voltage to 0.2 V improved removals to 76–96% and increased TOC removal to 64%.^90^ The electrical input to the electrodes was very low, 8.67 × 10^−3^ kW h m^−3^, but total energy reached 48.89 kWh m^−3^ when stirring and aeration were included.^90^ A Ti_4_O_7_/Ti_5_O_9_ reactive electrochemical membrane treating real secondary effluent achieved greater than 98% carbamazepine degradation and 70% TOC mineralization at 73 A m^−2^, 4.41 g h^−1^ m^−2^ TOC flux, and 0.74 kWh m^−3^; increasing current density raised the flux limit but also increased perchlorate from about 130 µg L^−1^ at 73 A m^−2^ to 25 mg L^−1^ at 290 A m^−2^.^76^ These two studies show that scale-relevant reactors can be efficient only within constrained windows.

**Fig. 8:**
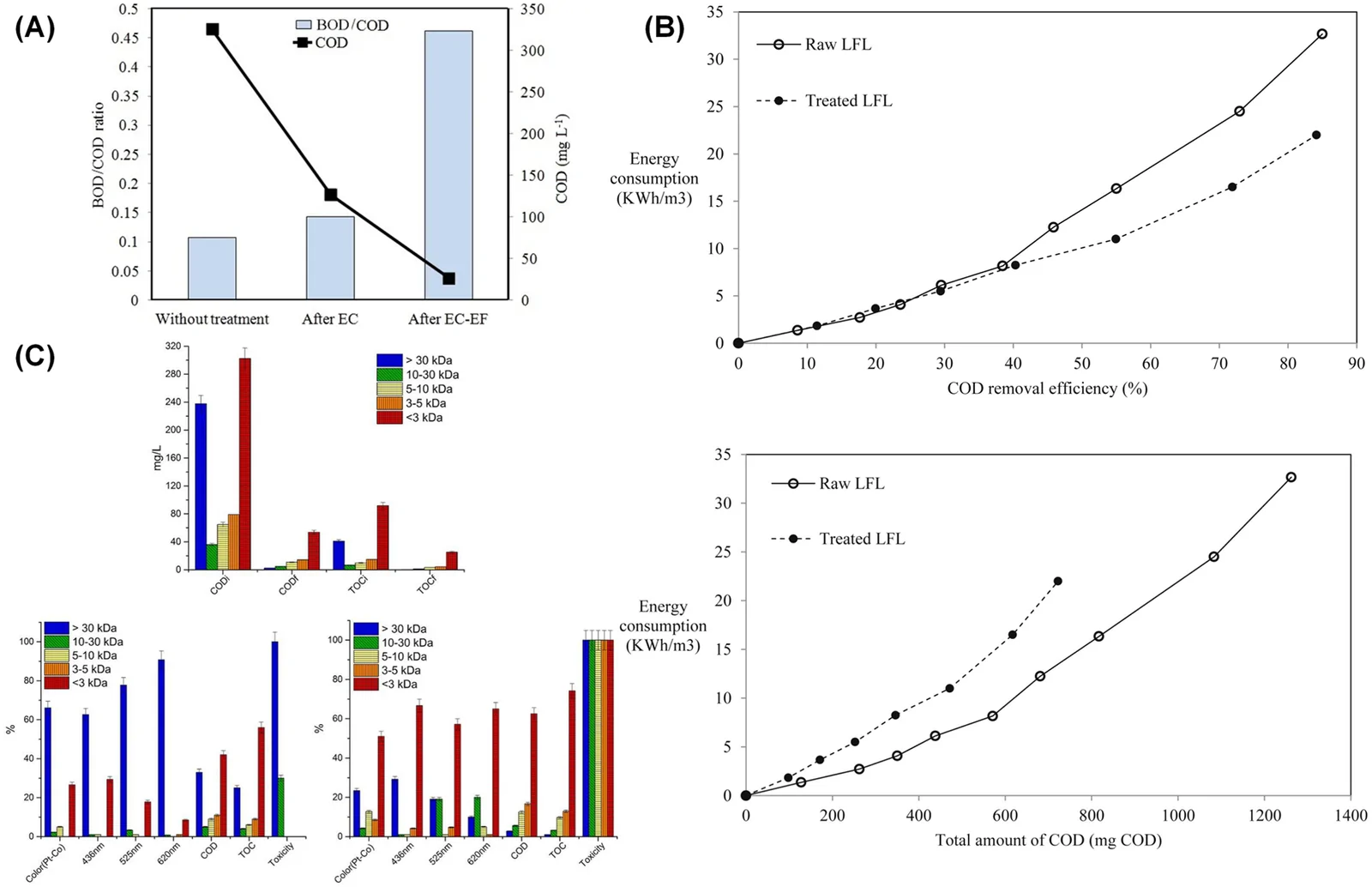
Real-wastewater and hybrid-treatment endpoints. (A) COD and biodegradability changes in textile wastewater.^103^. (B) Energy demand during landfill-leachate electro-oxidation.^105^. (C) Molecular-weight and toxicity changes requiring adsorption polishing.^111^

Application claims require endpoints beyond removal, and Fig. 8 links these endpoints to real-wastewater evidence. Fig. 8(A) shows COD and BOD/COD changes during textile treatment;^103^Fig. 8(B) relates landfill-leachate COD removal to energy consumption;^105^ and Fig. 8(C) connects molecular-weight redistribution with toxicity, explaining why activated-carbon polishing was needed.^111^

### 5.3 Energy, cost, scale-up, electrode lifetime, and regulatory translation

Energy and cost reporting remain the weakest part of EAOP translation. Values reported as kWh m^−3^, kWh kg_COD_^−1^, kWh kg_TOC_^−1^, kWh g^−1^, kWh log^−1^ m^−3^, Wh L^−1^, and operating cost per cubic metre are not interchangeable unless influent concentration, treatment endpoint, reactor volume, current efficiency, auxiliary energy, and chemical dosing are also reported. The contrast between electrode-only and total-system energy is especially important. In the 20 L bio-electro-Fenton study, the applied-voltage energy was almost negligible compared with aeration and stirring.^90^ In photoelectro-Fenton, lamp energy can dominate unless sunlight is used or the radiation field is optimized.^89^ In industrial pretreatment, a high energy input may still be justified when the electrochemical unit replaces a more expensive chemical process, as in the nitrobenzene pilot study where the pilot operating cost was reported as 18.8% of the replaced Fenton-flocculation unit.^112^

Electrode lifetime and secondary pollution also decide whether real applications are credible. PbO_2_-based anodes can be effective for nitrobenzene or other industrial wastewaters, but Pb leaching, coating lifetime, and spent-electrode management must be included in regulatory assessment.^112^ BDD and Magnéli-phase anodes are more stable in many oxidative windows, yet they can form chlorate and perchlorate in chloride-rich waters. Sacrificial iron or aluminum electrocoagulation improves removal of solids and color but generates sludge and shifts part of the treatment burden to solids handling. Carbonaceous cathodes and membranes may foul, wet, or lose activity in real effluents. These issues are not reasons to reject EAOPs; they are reasons to define the application boundary precisely.

Table 3 applies the same five-column comparison to authentic, spiked-real, and scale-relevant streams. The first column preserves source traceability; matrix and scale are separated from the treatment train; outcomes retain their reported basis; and the last column states missing toxicity, by-product, durability, or total-energy evidence.

**Table 3:** Application evidence matrix for EAOPs and hybrid systems in wastewater, with matrix scale, treatment window, endpoint package, and residual risk^a^

Ref.	Matrix and scale context	Treatment train and window	Comparable endpoint package	Transferability and risk
103	Matrix/scale: real textile effluent; COD 325 mg L^−1^, TOC 52 mg L^−1^	Sequential EC-EF; Fe EC followed by BDD/carbon felt EF; EC pH 8.75, 20 mA cm^−2^; EF pH 3, 10 mA cm^−2^; 60 + 60 min	Endpoints: COD 97%, TSS 98%, TOC/mineralization 97–98%; COD fell to 12 mg L^−1^; energy 0.45 kWh kg^−1^ removed TOC	Decision note: real wastewater batch; pH adjustment and sludge handling required; TN removal only 32%
97	Matrix/scale: tetracycline in synthetic wastewater-type dual chamber	Bio-electro-Fenton with carbon felt electrodes and FeSO_4_; catholyte pH 3; 24 h; TC 10 mg L^−1^; glucose anolyte	Endpoints: TC degradation 99.04%; anodic COD removal 85.71%; H_2_O_2_ 3.27 mg L^−1^; power density 93.13 mW m^−2^ at optimum	Decision note: synthetic matrix despite wastewater framing; slow, acidic, Fe-dosed operation
113	Matrix/scale: industrial PFAS wastewater; PFHxA 1240 µg L^−1^, TOC 12.7 mg L^−1^	BDD EO coupled with 185 nm VUV; 10 mA cm^−2^; pH 7.5 industrial wastewater; 20 h	Endpoints: all detected PFAS removed at least 96.2%; spiked PFOA defluorination increased from 28.5% in EO to 48.6% in EO + VUV	Decision note: real industrial PFAS sample; VUV lamp energy not normalized and short-chain PFAS accumulated early
107	Matrix/scale: four real petrochemical wastewaters; COD 20450–52300 mg L^−1^	Ru–Ir MMO electrooxidation; 64 A; 5.5, 4.0/4.1, or 11 V; 6 min	Endpoints: COD removal 79.1%, 54.56%, 57.3%, and 32.2%; *k* = 0.300–0.072 min^−1^; energy 85–235 kW h m^−3^	Decision note: real high-TDS industrial wastewater; COD-only endpoint and high energy intensity
114	Matrix/scale: real petroleum refinery wastewater	Graphite-aluminum anodic oxidation in batch recirculation; pH 3; 12 mA cm^−2^; 120 min	Endpoints: confirmatory COD removal averaged 96.1%; reported EEC range about 0.46–5.18 kWh m^−3^ across design table	Decision note: conference-scale evidence; wastewater characterization partly ambiguous; no toxicity/by-product data
106	Matrix/scale: raw tannery wastewater, high salinity	Continuous-flow EO with Ti/BDD, Ti/RuO_2_, or Ti/IrO_2_; 80 mA cm^−2^; 6 h for COD endpoint; up to 12 h for TOC	Endpoints: Ti/BDD COD removal 98.45%; Ti/RuO_2_ 70.15%; Ti/IrO_2_ 62.01%; Ti/BDD TOC removal nearly 70% after 12 h	Decision note: real flow cell; long electrolysis and chlorine oxyanion monitoring needed
112	Matrix/scale: nitrobenzene chemical-plant wastewater	Pilot electrooxidation with Ti/SnO_2_–Sb/Ce–PbO_2_ anode; 20 mA cm^−2^; HRT 90 min; 827.5 L; 30 d pilot	Endpoints: static pilot test: NB 96.2%, COD 68.8%; effluent NB 2.14–4.73 mg L^−1^; pilot cost 18.8% of replaced Fenton-flocculation	Decision note: strong scale evidence; PbO_2_ lifetime/leaching and residual COD 1106–1520 mg L^−1^ remain concerns
104	Matrix/scale: real textile wastewater; COD 1.10 gO_2_ L^−1^, TOC 0.45 g L^−1^	AO/BDD, EF/Pt, and EF/BDD comparison; 200 mA; EF pH 3; 6 h	Endpoints: TOC after 6 h: EF/Pt 52%, AO/BDD 75%, EF/BDD 95%; EF/BDD energy 18 kWh m^−3^ and 55 kWh kgTOC^−1^ at 4 h	Decision note: real textile matrix; best mineralization required pH 3 and Fe; BDD formed chlorate/perchlorate
90	Matrix/scale: six pharmaceuticals in synthetic and real secondary effluent	20 L continuous-flow bio-electro-Fenton; HRT 26 h; 0.1–0.2 V; Fe^2+^ 0.3 mM	Endpoints: real effluent removal at 0.2 V: 78–96% across six pharmaceuticals; TOC 64%; total energy 48.89 kWh m^−3^	Decision note: scale-relevant 20 L reactor; aeration/stirring dominated energy and real matrix reduced performance
76	Matrix/scale: carbamazepine in real secondary effluent	Ti_4_O_7_/Ti_5_O_9_ reactive electrochemical membrane; 73 A m^−2^; TOC flux 4.41 g h^−1^ m^−2^; natural pH 7.5	Endpoints: >98% CBZ degradation; 70% TOC mineralization; energy 0.74 kWh m^−3^; perchlorate about 130 µg L^−1^ at 73 A m^−2^	Decision note: strong flow-through evidence; higher current raised throughput but sharply increased perchlorate
93	Matrix/scale: real soluble coffee effluent; COD 3.465 g L^−1^, TOC 1.301 g L^−1^	Sequential EC-EO; EC 62.33 min, EO 53 min; pH 7.98; J_EC 149.23 A m^−2^, J_EO 500 A m^−2^	Endpoints: COD reduction 73.90% in verification; TOC 63.5%; BOD_5_/COD rose to about 0.6; cost 8.90 USD m^−3^	Decision note: real industrial effluent; NaCl addition and halogenated by-product risk not quantified
111	Matrix/scale: real denim textile wastewater; COD 720 mg L^−1^, TOC 164 mg L^−1^	EC + EO + activated carbon; EC 10 min at 5 mA cm^−2^; EO 30 min at 10 mA cm^−2^; AC 3.7 min	Endpoints: EC + EO: 100% color, 88% COD, 79% TOC; full train cost 3.83 USD m^−3^; AC reduced Artemia mortality to 0%	Decision note: toxicity persisted after EC + EO and required adsorption polishing
75	Matrix/scale: secondary effluents spiked with acetaminophen	EF/AO with Ti_4_O_7_ anode and carbon felt cathode; pH 3; 250 mA; 20 min parent endpoint; 8 h carbon/toxicity endpoint	Endpoints: >99% ACT removal after 20 min; TOC after 8 h: 90%, 76%, and 46% in three effluents; energy for parent removal 1.1–2.0 kWh m^−3^	Decision note: parent removal required much less energy than mineralization/toxicity control
96	Matrix/scale: parabens in real secondary treated effluent	Solar photoelectro-Fenton with BDD or RuO_2_ anode; pH 3; 10 mA cm^−2^; 150–240 min	Endpoints: BDD removed all parabens in 150 min and reached 66% TOC at 240 min; RuO_2_ reached >95% in 180 min and 47–52% TOC	Decision note: pre-pilot solar plant; low-current operation reduced but did not eliminate chlorination
105	Matrix/scale: raw and MBR-treated landfill leachate; seasonal comparison	MBR followed by BDD electrooxidation; MBR SRT 80 d; EOP 3 A, 120 min; pH 8.4	Endpoints: EOP COD removal 84.2% summer and 56.6% winter; TOC 71.6% and 37.1%; DEHP 100% and 70.6%; energy 16–22 kW h m^−3^	Decision note: pilot/lab sequential system; Daphnia toxicity increased after EOP

a Comparison note: *C*_0_ denotes initial concentration and NR denotes not reported. Source units are retained; missing values are not inferred. The final column identifies the endpoint that limits comparison or deployment.

The strongest Table 3 entries combine real matrices, carbon endpoints, energy, and toxicity or by-product information. The weakest report only COD or parent removal. EAOP deployment should therefore be assigned to one of four roles: destruction of a concentrated stream, pretreatment before biology, post-treatment of micropollutants, or polishing after coagulation or adsorption. Table 4 makes the five comparison metrics and their mandatory context explicit so that studies can be interpreted without converting incompatible units or inferring unreported values.

**Table 4:** Standardized five-metric reporting basis for cross-study EAOP comparison

Metric	Normalized reporting basis	Mandatory context	Decision meaning
Removal rate	*C*/*C*_0_*versus* time; kobs only when the kinetic model is justified	*C*_0_, sample volume, time, matrix, electrode area	Separates rapid parent loss from loading and mass-transfer effects
Mineralization	TOC or COD removal at the same endpoint; element balance when relevant	Initial TOC/COD, treatment time, inorganic-carbon handling	Tests whether parent disappearance progresses toward carbon conversion
Energy	kWh m^−3^ or EE/O for a defined endpoint; auxiliary energy reported separately	Cell voltage, current, volume, aeration, pumping, light, pressure	Prevents electrode-only energy from being presented as system energy
By-products/toxicity	Target oxyhalides/AOX plus non-target products and a quenched bioassay	Halides, pH, oxidant residual, sampling and quenching method	Determines whether oxidation lowers risk rather than redistributing it
Matrix adaptability	Performance retention from synthetic to spiked-real to authentic wastewater	Conductivity, chloride, alkalinity, NOM, solids, co-contaminants	Defines the transferable operating window and treatment-train role

## 6. Conclusions, technology roadmap, and future perspectives

This review's principal contribution is a causal framework that connects mechanism, material, matrix, and implementation. Across anodic oxidation, electro-Fenton chemistry, mediated oxidation, and reactive membranes, treatment performance emerges from the complete system rather than from electrode identity alone. The framework also explains why a material that performs well in sulfate electrolyte can fail in authentic wastewater, and why rapid parent loss can coexist with poor mineralization or elevated toxicity.

The quantitative synthesis supports three decision rules. First, current density or potential must be reported with initial load, electrode area, volume, residence time, and matrix composition. Second, parent removal must be paired with carbon conversion and a class-specific endpoint such as toxicity, defluorination, dechlorination, or oxyhalide formation. Third, electrical input to the cell must be distinguished from total-system energy for aeration, pumping, mixing, pressure, irradiation, and reagent production. The five-metric basis in Table 4 makes these rules operational.

The main barriers to translation are now identifiable rather than generic: oxidant scavenging and competing reactions in real matrices; chlorinated or brominated products and oxyhalides; transient toxicity during partial oxidation; catalyst leaching, electrode passivation, membrane fouling, and gas-diffusion-electrode wetting; incompatible energy denominators; and short operating campaigns that do not establish lifetime. These gaps define the minimum evidence needed before an EAOP can be compared with ozonation, adsorption, membranes, or biological treatment.

Near-term research should combine *operando* electrochemical and spectroscopic measurements with high-resolution target and non-target mass spectrometry, fluorine or halogen mass balances, and effect-based bioassays. This combination can identify when a change in current, pH, halide concentration, or residence time moves the process from productive oxidation into a by-product or toxicity window. Materials studies should report leaching, regeneration, fouling, and performance loss over extended operation, including the fate of spent catalysts and electrodes.

**Fig. 9:**
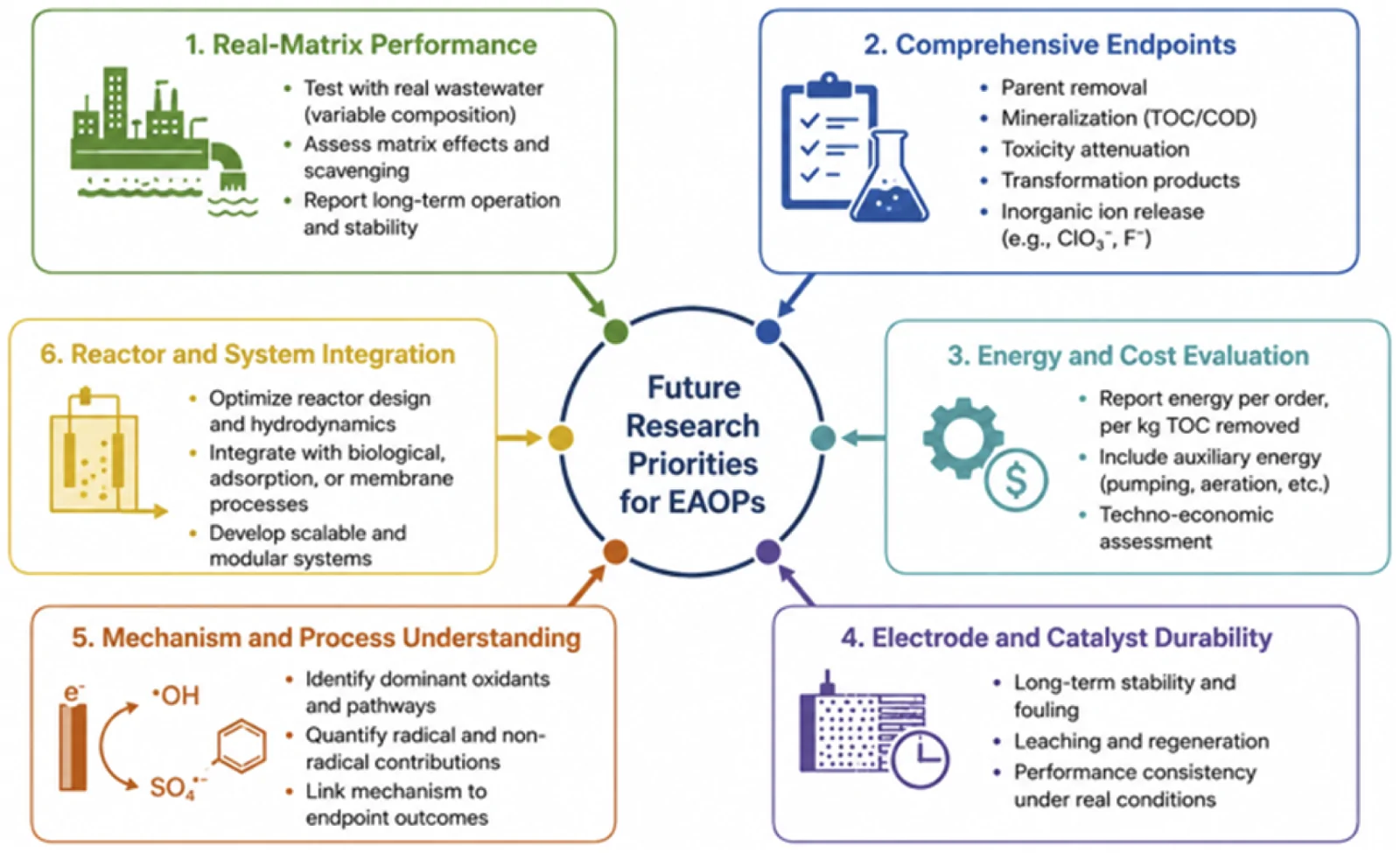
Stage-gated technology roadmap for EAOP deployment. The stages connect mechanistic validation, complete endpoint measurement, durability, real-matrix testing, normalized energy and cost, modular treatment-train integration, and data-assisted control. Advancement to the next stage requires explicit pass criteria rather than a removal-percentage target.

Medium-term development should integrate modular flow-through or falling-film reactors, renewable power, selective adsorption or membrane concentration, and biological polishing. Data-assisted control can then be used for a defined purpose: soft sensors should estimate oxidant exposure and toxicity risk, model-predictive control should adjust current and flow to a specified endpoint, and digital twins should include auxiliary energy, electrode ageing, and by-product constraints rather than optimize removal alone. Fig. 9 organizes these priorities as a stage-gated technology roadmap from mechanistic proof to authentic wastewater, long-term operation, techno-economic analysis, life-cycle assessment, and regulatory monitoring.

In conclusion, EAOPs are most credible when assigned a defined role within a treatment train and operated inside a verified chemical and engineering window. Their distinctive advantage is tunable *in situ* oxidant generation; their central risk is interpreting analytical disappearance as environmental safety. Applying the mechanism-material-matrix-implementation framework, the standardized endpoint set, and the stage-gated roadmap can move the field from isolated high-removal demonstrations toward reproducible, energy-transparent, and risk-controlled wastewater treatment.

## Author contributions

Z. Z. contributed to conceptualization, methodology, formal analysis, visualization, and writing – original draft. G. J. and Q. L. contributed to literature investigation, data curation, and validation of the quantitative evidence. N. Z. contributed to evidence interpretation and writing – review and editing. Z. C. contributed to conceptualization, supervision, project administration, funding acquisition, and writing – review and editing. All authors have read and agreed to the published version of the manuscript.

## Conflicts of interest

There are no conflicts to declare.

## Data Availability

No new experimental data were generated in this review.
